# Cold and warmth intensify pain-linked sodium channel gating effects and persistent currents

**DOI:** 10.1085/jgp.202213312

**Published:** 2023-08-02

**Authors:** Sophia Kriegeskorte, Raya Bott, Martin Hampl, Alon Korngreen, Ralf Hausmann, Angelika Lampert

**Affiliations:** 1 University Hospital, RWTH Aachen University, Institute of Neurophysiology, Aachen, Germany; 2 Nanion Technologies GmbH, München, Germany; 3 The Leslie and Susan Gonda multidisciplinary Brain Research Center, Bar Ilan University, Ramat Gan, Israel; 4 The Mina and Everard Goodman Faculty of Life Sciences, Bar Ilan University, Ramat Gan, Israel; 5 University Hospital, RWTH Aachen University, Institute of Clinical Pharmacology, Aachen, Germany

## Abstract

Voltage-gated sodium channels (Na_v_) are key players in excitable tissues with the capability to generate and propagate action potentials. Mutations in the genes encoding Na_v_s can lead to severe inherited diseases, and some of these so-called channelopathies show temperature-sensitive phenotypes, for example, paramyotonia congenita, Brugada syndrome, febrile seizure syndromes, and inherited pain syndromes like erythromelalgia (IEM) and paroxysmal extreme pain disorder (PEPD). Nevertheless, most investigations of mutation-induced gating effects have been conducted at room temperature, and thus the role of cooling or warming in channelopathies remains poorly understood. Here, we investigated the temperature sensitivity of four Na_v_ subtypes: Na_v_1.3, Na_v_1.5, Na_v_1.6, and Na_v_1.7, and two mutations in Na_v_1.7 causing IEM (Na_v_1.7/L823R) and PEPD (Na_v_1.7/I1461T) expressed in cells of the human embryonic kidney cell line using an automated patch clamp system. Our experiments at 15°C, 25°C, and 35°C revealed a shift of the voltage dependence of activation to more hyperpolarized potentials with increasing temperature for all investigated subtypes. Na_v_1.3 exhibited strongly slowed inactivation kinetics compared with the other subtypes that resulted in enhanced persistent current, especially at 15°C, indicating a possible role in cold-induced hyperexcitability. Impaired fast inactivation of Na_v_1.7/I1461T was significantly enhanced by a cooling temperature of 15°C. The subtype-specific modulation as well as the intensified mutation-induced gating changes stress the importance to consider temperature as a regulator for channel gating and its impact on cellular excitability as well as disease phenotypes.

## Teaser

Kriegeskorte et al. show that activation of the sodium channel subtypes Na_v_1.3, Na_v_1.5, Na_v_1.6, and Na_v_1.7, and two pain-linked mutations is alleviated by increasing temperature. Cooler temperatures, on the other hand, strongly enhance persistent currents of Na_v_1.3. The impaired fast inactivation of the pain-linked Na_v_1.7/I1461T mutation is further impaired by cooling, mimicking clinical findings.

## Introduction

Voltage-gated sodium channels (Na_v_) play an essential role in the electrical signaling of cells. With their capability to activate and inactivate rapidly in response to changes in membrane voltage, they are initiating the upstroke of action potentials and are key players in excitable tissues. Nine Na_v_ α-subunits, Na_v_1.1–1.9, and four auxiliary β-subunits have been identified in humans so far (Catterall et al., 2005; Catterall, 2000; Morgan et al., 2000; Yu et al., 2003). Na_v_1.1, Na_v_1.2, Na_v_1.3, and Na_v_1.6 are mainly, but not exclusively, expressed in the central nervous system (CNS; Liang et al., 2021; Whitaker et al., 2000; Vacher et al., 2008). Na_v_1.4 is mainly expressed in skeletal and Na_v_1.5 in cardiac muscles (Rogart et al., 1989; Trimmer et al., 1989), while Na_v_1.7, Na_v_1.8, and Na_v_1.9 are found in the peripheral nervous system (PNS; Bennett et al., 2019; Fukuoka et al., 2008).

The voltage dependence of activation and inactivation, their time constants as well as the recovery from inactivation are, like all cellular processes, influenced by temperature changes. Nevertheless, comprehensive studies characterizing temperature-dependent gating are rare. Because Na_v_s are an important factor for the overall excitability of neurons, serious functional consequences are related to temperature-induced changes in their gating properties. For example, the enhanced functionality of Na_v_1.2 at febrile (40–41°C) compared with physiological (36°C) temperature mediated the increase in neuronal excitability in in vitro experiments with cortical tissue (Ye et al., 2018). Computer simulations revealed that already small elevations in temperature from physiological to fever conditions increase the excitability of central neurons expressed in higher firing rates and faster action potential conduction velocity (Ye et al., 2018). In vivo experiments with mice, which were exposed to a 42°C environment for 30 min, showed that febrile temperature alone can provoke seizure-related behavioral changes (Ye et al., 2018). Moreover, gain or loss of function mutations in Na_v_s causes severe, temperature-provoked diseases, like Brugada syndrome (Samani et al., 2009; Keller et al., 2005), Paramyotonia congenita (PMC; Bouhours et al., 2004; Carle et al., 2009; Ke et al., 2017), febrile epileptic syndromes (Volkers et al., 2013; Peters et al., 2016), and inherited pain syndromes (Körner and Lampert, 2020).

Na_v_s expressed in peripheral nerve endings of the skin are necessary to generate and propagate action potentials encoding sensory information. Compared with Na_v_s of the CNS, they are exposed to much larger variations in temperature. While the temperature in the body core is held stably at ∼37°C, skin temperature of the extremities like hand or feed can drop to values of 17°C and lower when exposed to a cold environment, for example ice-cold water (Dupuis, 1987; Isii et al., 2007). The tetrodotoxin (TTX)-resistant Na_v_1.8 is necessary to transduce nociceptive information in sensory neurons at low temperatures in mice (Zimmermann et al., 2007). While at 10°C, TTX-sensitive Na_v_s (mainly Na_v_1.7 in the PNS) are mostly slowly inactivated at the resting membrane potential, Na_v_1.8 remains excitable. To encode noxious heat, the TTX-resistant Na_v_1.9 is required in rodents and undergoes a large gain of function with increasing temperature (Touska et al., 2018). Thus, it is likely that different Na_v_ isoforms have different intrinsic sensitivity to temperature changes.

Rare inherited chronic pain syndromes with temperature-sensitive phenotypes can be caused by mutations in Na_v_1.7 (Körner and Lampert, 2020). Inherited erythromelalgia (IEM), which is characterized by burning pain, redness, and warmth of the extremities, is in most patients typically provoked by elevated ambient temperature and exercise, while only extensive cooling brings relief (van Genderen et al., 1993). In contrast, mutations causing paroxysmal extreme pain disorder (PEPD) lead to attacks of rapidly developing burning pain in regions of the rectum, eye, and mandibular, which may be accompanied by vegetative symptoms (Bennett et al., 2019). In this pain syndrome cooling, for example, cold wind is described as one possible trigger factor (Fertleman et al., 2007). Most of the known IEM mutations cause shifts in the voltage dependence of half-maximal (*V*_*1/2*_) activation to more hyperpolarized potentials, while the PEPD mutations mainly impair steady-state fast inactivation (Baker and Nassar, 2020; Tang et al., 2015; Jarecki et al., 2008). The effects observed have been linked to neuronal hyperexcitability causing pain. Here, we focus exemplary on the IEM-linked mutation Na_v_1.7/L823R that inserts an additional positive charge in the voltage sensor of domain II (DII; Lampert et al., 2009), and the PEPD mutation Na_v_1.7/I1461T, changing the unipolar isoleucine of the inactivation-motif to a polar threonine (Fertleman et al., 2006).

Neuropathic pain, for example, after a nerve or spinal cord injury, is often accompanied by cold allodynia in which already innocuous cold stimuli lead to pain (Jensen and Finnerup, 2014). Thermosensitive transient receptor potential ion channels (TRPs) have been identified to enable somatosensory neurons to sense temperature (Caterina et al., 1997; Tominaga et al., 1998), with TRPM8 being sensitive to cool temperatures (McKemy, 2005; McKemy et al., 2002) and necessary for cold allodynia (Caspani et al., 2009; Knowlton et al., 2013; Colburn et al., 2007). But there is also evidence that Na_v_s are crucial for cold-induced pain in different neuropathic pain conditions (Sittl et al., 2012; Zimmermann et al., 2013).

Despite the temperature sensitivity of several Na_v_-channelopathies, information about temperature-induced changes in gating properties is mostly lacking. Most electrophysiological experiments, due to the technical challenges at physiological temperature, are conducted at room temperature, and reliable data of electrophysiological characterizations at different temperatures are rare. A few studies have investigated the effect of temperature on different Na_v_ subtypes. Most of them revealed that with increasing temperature, the speed of gating, reflected in the time constants, is accelerated, while the effect on the voltage dependence of steady-state activation and inactivation is still under debate and varies depending on the experimental conditions (Ruff, 1999; Sarria et al., 2012; Thomas et al., 2009; Ye et al., 2018; Zimmermann et al., 2007; Egri et al., 2012). Only a few studies comparing different subtypes with regard to their temperature sensitivity under uniform conditions have been conducted so far, and many of them suffer from a limited number of experiments (Touska et al., 2018; Zimmermann et al., 2007; Ye et al., 2018).

In this study, we investigated four different Na_v_ subtypes, Na_v_1.3, Na_v_1.5, Na_v_1.6, and Na_v_1.7, regarding their temperature-dependent gating and to evaluate a possible subtype-specific modulation. Na_v_s expressed, e.g., in cold-sensing neurons have to conduct stable action potentials at lowered temperatures, while those in heat-sensing neurons must work properly at elevated temperatures. We hypothesize that Na_v_ subtypes display specific temperature dependence and that they thus show distinct expression patterns. In this study, we focus on the first part of this hypothesis using high throughput, highly comparable and reliable patch clamp recordings of wild-type (WT) Na_v_s. Furthermore, we studied two mutations of Na_v_1.7, causing IEM and PEPD to test whether the clinical phenotype is reflected in their gating properties. We performed high-throughput patch clamp experiments with an automated patch clamp device, the SyncroPatch 384, at 15°C, 25°C, and 35°C, which revealed a subtype-specific modulation by temperature as well as intensified mutation-induced gating changes. The voltage dependence of activation was shifted to more hyperpolarized potentials with increasing temperature for all investigated subtypes, with an enhanced left shift in the IEM mutation, while *V*_*1/2*_ of steady-state fast inactivation was in general less affected. Our results stress the importance to consider temperature as a regulator for channel gating and its impact on cellular excitability and disease phenotype.

## Materials and methods

### Cell culture and cell preparation

All investigated Na_v_ subtypes (rat [r] Na_v_1.3, human [h] Na_v_1.5, mouse [m] Na_v_1.6, hNa_v_1.7, hNa_v_1.7/L823R, and hNa_v_1.7/I1461T) were stably expressed in cells from the human embryonic kidney cell line HEK293 and kept under standard culture conditions (37°C and 5% CO_2_). Cell culture media and supplements are listed in Table S1.

All electrophysiological experiments were performed with cells at passage numbers lower than 30 and after reaching a confluency of 70–95%. 1 d prior to the recording, the media was refreshed, and the cells were incubated at 32°C and 5% CO_2_.

In order to carry out the experiments, the cells were washed twice with PBS-EDTA (PBS: PAN-Biotech; EDTA: Sigma-Aldrich), then treated with Trypsin/EDTA (Sigma-Aldrich), and then incubated at 37°C for 6 min to detach, before “External –Mg^2+^ –Ca^2+^” (Nanion Technologies GmbH) containing (in mM) 10 Hepes, 140 NaCl, 5 glucose, and 4 KCl was added. Subsequently, the cells were stored at 4°C for 10 min. Cells were pipetted up and down 8–10 times using fire-polished glass pipettes to break up cell clumps, External –Mg^2+^ –Ca^2+^ was added until a final volume of 30 ml and cells were transferred to the “Cell Hotel” on the SyncroPatch 384 (Nanion Technologies GmbH), where they rested at least 30 min at 10°C and shaking speed of 333 rpm before the recording was started.

### Electrophysiology

**Figure S1.**
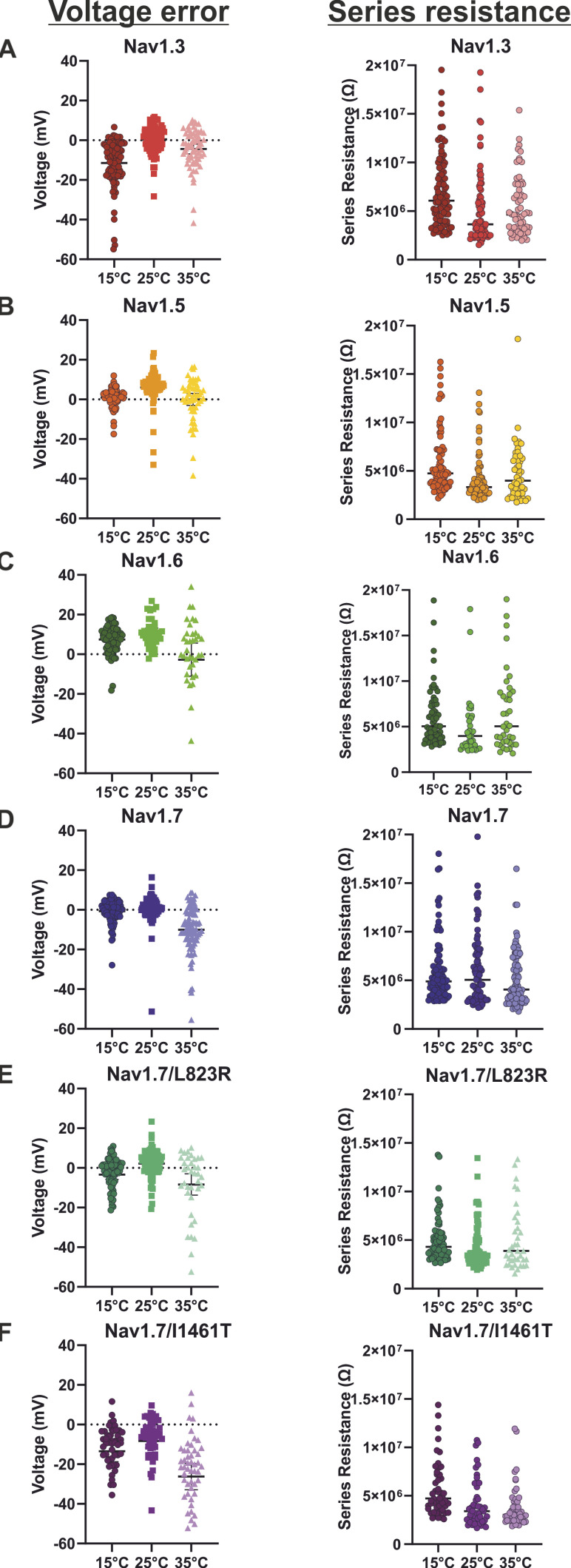
**Voltage error and series resistance. (A–F)** Voltage error (mV) and series resistance (Ω) of all tested Na_v_-subtypes. Data presented as mean ± 95% confidence interval. *n*-values are the same as for the activation parameter (Table 1).

Whole-cell voltage-clamp recordings at 15°C, 25°C, and 35°C were performed using the high-throughput patch clamp robot SyncroPatch 384 with “NPC-384T 1× S-Type” chips (2 µm holes, Nanion Technologies GmbH) and, for data acquisition, PatchControl384 Version 1.9.7 (Nanion Technologies GmbH). Whole-cell recordings were conducted according to Nanion’s procedure, including initialization, cell-catch, sealing, whole-cell formation, liquid application, and recordings. After achieving the whole-cell configuration, capacitive transients were canceled, series resistance compensation was set to 65%, leak currents were subtracted online using a P/4 procedure, and signals were sampled at 20 kHz. With an increase in temperature of 10°C, the liquid junction potential is shifted by 0.81 mV (liquid junction potential was calculated according to the stationary Nernst–Planck equation [Marino et al., 2014 *Preprint*] using LJPcalc software [https://swharden.com/LJPcalc]). Because of its small size, this shift was not corrected during the experiments. Detailed information regarding series resistance and voltage error are shown in Fig. S1 and Table S2.

The integrated temperature control unit of the SyncroPatch 384 allowed the adjustment of the temperature in a range from 10°C to 43°C throughout the experiment. The temperature of both the solution and measurement chamber was set to either 15°C, 25°C, or 35°C. For each subtype and temperature, one experiment (384 wells) on the SyncroPatch was performed. Experiments at different temperatures were performed individually so that each cell was only recorded at one temperature. Cells of the same preparation were measured at several temperatures on the same day to increase comparability.

The intracellular solution “Internal CsF110” contained (in mM) 10 EGTA, 10 Hepes, 10 CsCl, 10 NaCl, and 110 CsF. The extracellular solution “External Standard” (only used for recordings of Na_v_1.6) contained (in mM) 140 NaCl, 10 Hepes, 5 glucose, 4 KCl, 2 CaCl_2_, and 1 MgCl_2_, while the extracellular solution “External NMDG 60” (used for the recordings of all other channels) contained (in mM) 80 NaCl, 60 NMDG, 10 Hepes, 5 glucose, 4 KCl, 2 CaCl_2_, and 1 MgCl_2_ (all solutions made by Nanion Technologies GmbH).

The channels’ activation was assessed using 30 ms pulses to a range of test potentials (−85 to + 30 mV) from the resting membrane potential of −120 in 5 mV steps with an interval of 5 s at resting membrane potential between each sweep. The conductance–voltage (G-V) relationship, the inactivation time constant *τ*, as well as the persistent currents, were determined from these recordings. The voltage-dependent Na_v_ conductance *G*_*Na*_ was calculated according to

$$G_{Na}=\frac{I_{Na}}{V_{m}-E_{rev}}\text{,}$$

where *I*_*Na*_ is the peak current at the voltage *V*_*m*_ and *E*_*rev*_ is the reversal potential for sodium, determined for each cell individually. Activation curves were derived by plotting *G*_Na_ normalized to the maximum conductance *G*_*Na,max*_ as a function of test potential and fitting it with the Boltzmann distribution equation:

$$G_{Na}=\frac{G_{Na,\max}}{1+e^{\frac{V_{1/2}-V_{m}}{k}}}\text{,}$$

where *V*_*1/2*_ is the membrane potential at half-maximal activation, *V*_*m*_ is the membrane voltage, and *k* is the slope factor. Persistent sodium current, *I*_*pers*_, was defined as the mean remaining current between 26.5 and 29.5 ms for each 30 ms activation pulse and normalized to the peak current *I*_*peak*_. It is plotted as a function of test potential.

The population average of all included traces was used to determine the inactivation time constant *τ* with a mono-exponential fit on the decayed part of these traces. To quantitatively determine the dependence of the inactivation process on temperature, the Arrhenius equation was described as follows:

$$\alpha =e^{-z\left(\alpha \right)V}\times e^{-\frac{E_{a}\left(\alpha \right)}{RT}}\text{,}$$

where *α* is the forward rate constant in the transition between open and inactivated state, O⇄I, *z* is a proportionality constant, *E*_*a*_ is the activation energy, *R* is the gas constant, *T* is the absolute temperature, and V is the applied voltage. β describes the backward rate constant in the transition between open and inactivated state. Since *τ ≈ 1/α* at depolarized potentials (where *Ea*(*α*) ≈ *Ea*(*β*)), it was possible to use the Arrhenius plot to estimate *E*_*a*_ by plotting ln(τ) as a function of *1/T* because the slope corresponds to *E*_*a*_*/R* in this case. The plot of *E*_*a*_ as a function of voltage will be parallel to the voltage axis. At more negative potentials (where *Ea*(*α*) ≠ *Ea*(*β*)), *τ = 1/*(α + β), and therefore the simple Arrhenius analysis is generally not applicable. The plot will deviate from a horizontal line.

The voltage dependence of steady-state fast inactivation was measured using a series of 500-ms prepulses from −130 to −20 mV in 10-mV steps followed by a 40-ms test pulse to 0 mV that assessed the non-inactivated transient current. The normalized peak inward currents were fitted using a Boltzmann distribution equation:

$$\frac{I_{Na}}{I_{Na,\max}}=\frac{1}{1+e^{\frac{V_{1/2}-V_{m}}{k}}}\text{,}$$

where *I*_*Na,max*_ is the peak sodium current elicited after the most hyperpolarized prepulse, *V*_*m*_ is the preconditioning pulse potential, *V*_*1/2*_ is the half-maximal sodium current, and *k* is the slope factor.

The recovery from fast inactivation was measured using a 500-ms prepulse to 0 mV followed by a hyperpolarizing recovery pulse to −100 mV of varying duration (1–2,000 ms). Due to the amplifier settings, it was not possible to measure time intervals shorter than 1 ms. After that, another depolarizing test pulse to 0 mV was applied to assess the rate of recovered channels. The maximum inward current of the test pulse *I*_*recov*_ was normalized to the maximum inward current of the prepulse *I*_*pre*_ and plotted against the duration of the recovery pulse. The following double-exponential equation was used:

$$I=I_{plateau}+\propto_{fast}e^{\frac{-t}{\tau_{fast}}}+\propto_{slow}e^{\frac{-t}{\tau_{slow}}}\text{,}$$

where *I* is the current amplitude, *I*_*plateau*_ is the amplitude at recovery time t = 1 ms, *α*_*fast*_ and *α*_*slow*_ are the amplitudes for time constants *τ*_*fas*t_ and *τ*_*slow*_, and *t* is time*. %*_*fast*_ is the fraction of the overall recovery that is accounted for by the faster-recovering component following the equation:

$${\%}_{fast}=\frac{\propto_{fast}}{\left(I_{plateau+\alpha fast+\alpha slow}\right)}\times 100\%\text{.}$$

To evoke ramp currents, slow depolarizing pulses were applied from a holding potential of −120 to +5 mV or to +20 mV. Rates of 1.4, 2.5, and 5 mV/ms were investigated and the maximum inward current *I*_*ramp*_ was normalized to the maximum inward current elicited in the G-V-relationship *I*_*Act*_.

5-ms voltage pulses to 0 mV from the holding potential of −120 mV were applied at frequencies of 20, 50, and 100 Hz to assess the use-dependent current decay. The peak inward current of the 10th action potential *I*_*10th*_ was normalized to the peak inward current of the first action potential *I*_*1st*_.

### Data analysis and statistics

The recorded data were analyzed using DataControl384 version 2.0.0 (Nanion Technologies GmbH), IgorPro (WaveMetrics), and Prism version 9 (GraphPad Software).

For statistical testing, comparisons between two experimental groups were done by Student’s *t* test or a Mann–Whitney test in case of nonparametric testing. Comparisons between more than two experimental groups were done by one-way ANOVA followed by Sidak’s multiple comparison test for parametric testing and Kruskal–Wallis test with Dunn’s multiple comparisons in case of non-parametric testing. Statistical significance was defined as P < 0.05. All values are mentioned as mean ± SEM unless otherwise stated.

### Online supplemental material

**Figure S2.**
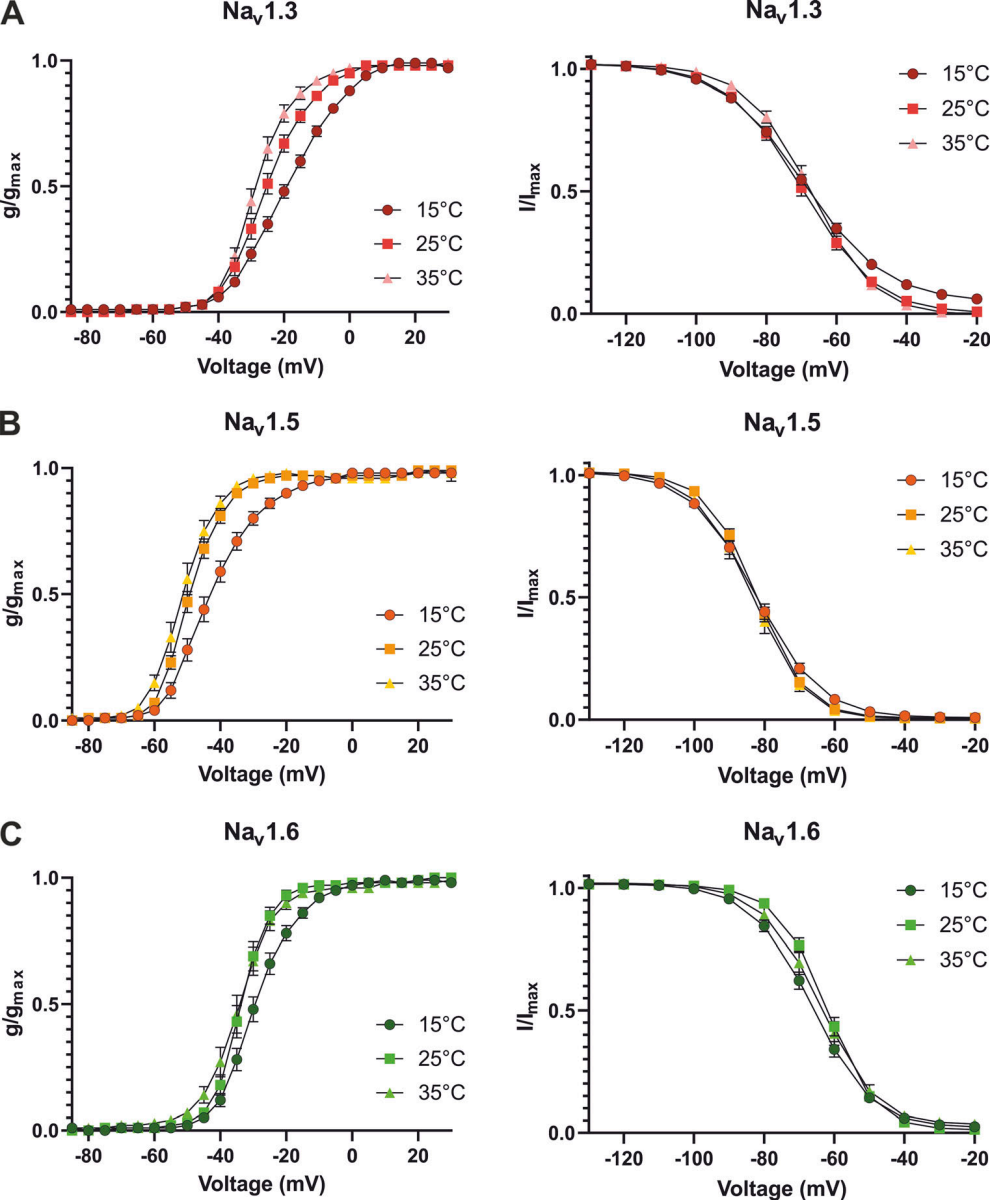
**Elevating temperature shifts the voltage dependence of activation but not the steady-state fast inactivation to more hyperpolarized potentials. (A–C)** Activation G-V-curve (left) and steady-state fast inactivation I-V curve (right) for (A) Na_v_1.3, (B) Na_v_1.5, and (C) Na_v_1.6. Data are shown as means ± 95% confidence interval.

Detailed information concerning the cell culture media and supplements for each cell line individually are listed in Table S1. Fig. S1 and Table S2 show the voltage error and series resistance for each Na_v_ subtype. Activation G-V curves and steady-state fast inactivation I-V curves of Na_v_1.3, Na_v_1.5, and Na_v_1.7 are shown in Fig. S2.

## Results

### Voltage dependence and kinetics of Na_v_ activation are modulated by temperature

**Figure 1.**
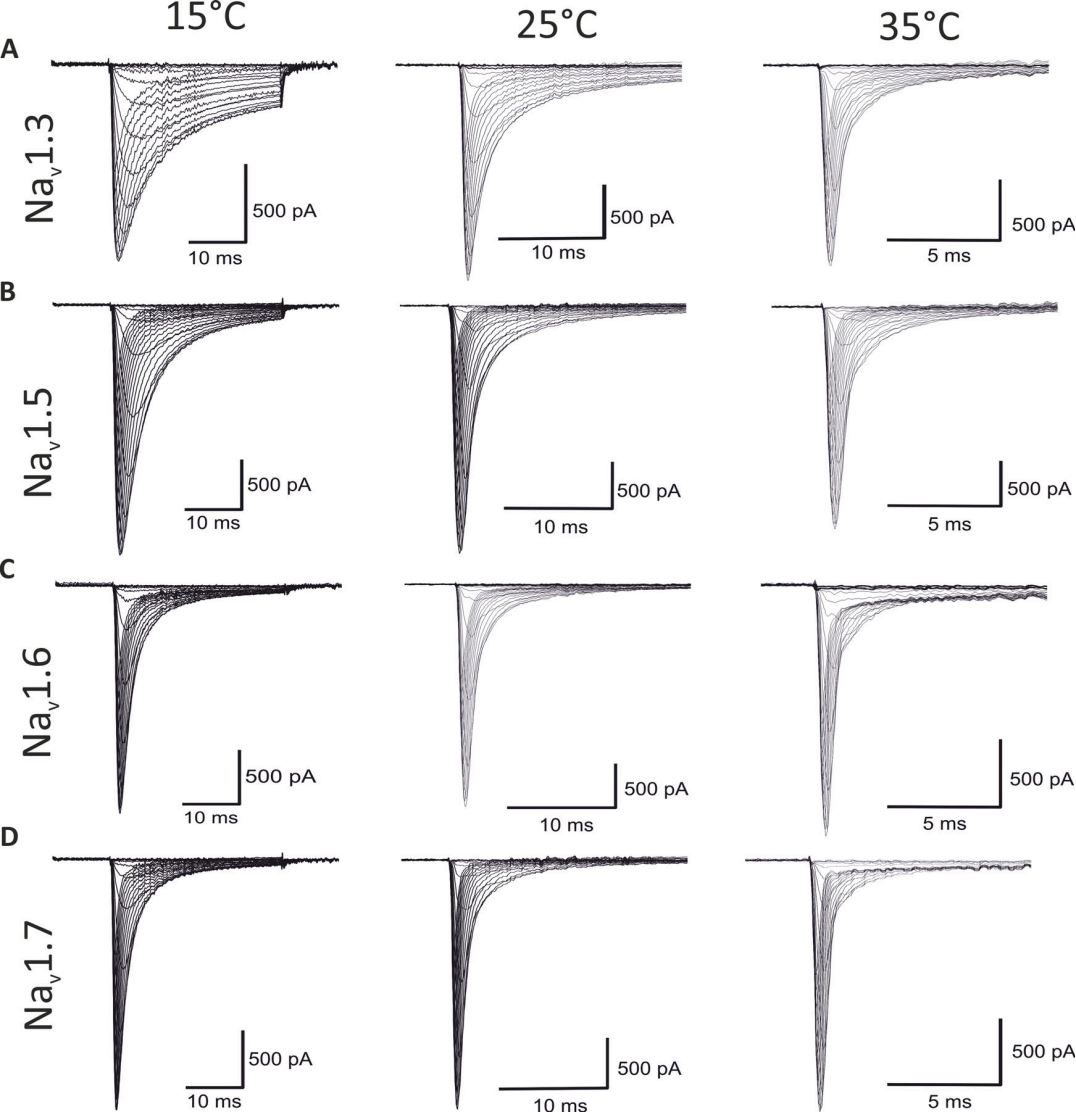
**Population average of all analyzed experiments.** Average of all included current traces, elicited by applying 30-ms depolarizing pulses from a holding potential of −120 in 5 mV steps from −85 to 30 mV. **(A–D)** Current traces of (A) Na_v_1.3, (B) Na_v_1.5, (C) Na_v_1.6, and (D) Na_v_1.7 at 15°C, 25°C, and 35°C. By presenting the average of all included recordings, a human selection bias that selects unusually nice recordings as representative traces was avoided. Note the higher time scale with increasing temperature.

The gating of Na_v_s is sensitive to temperature. Here, we investigated four WT isoforms: Na_v_1.3, Na_v_1.5, Na_v_1.6, and Na_v_1.7, each at 15°C, 25°C, and 35°C. Fig. 1 shows traces presenting the population average of all analyzed experiments.

**Figure 2.**
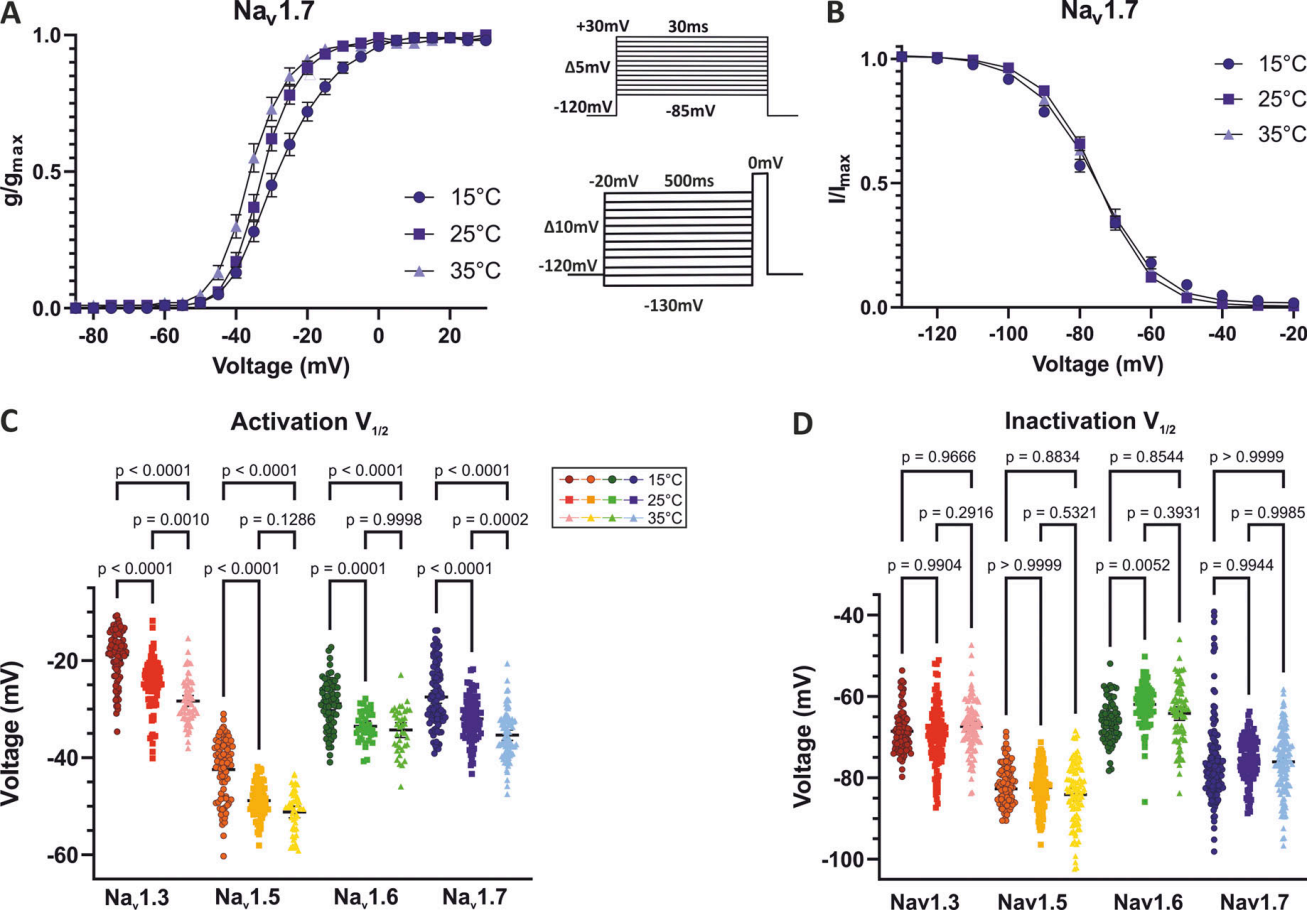
**Increased temperature shifts the voltage dependence of activation but not the steady-state fast inactivation to more hyperpolarized potentials.** The activation voltage dependence was shifted to more hyperpolarized potentials with elevating temperature from 15°C to 35°C for all tested channel isoforms. **(A)** Exemplary G-V curve elicited from Na_v_1.7 activation and **(B)** I-V curve of Na_v_1.7 steady-state fast inactivation. Voltage protocols in the inlet. **(C and D)** Values of half-maximal voltage dependence (*V*_*1/2*_) obtained from Boltzmann fit for individual traces. Means ± 95% confidence interval. One-way ANOVA with Sidak’s multiple comparisons test, exact P values are indicated.

First, we quantified the impact of temperature on Na_v_ activation. Without exception, we found hyperpolarizing shifts of *V*_*1/2*_ in the range between 5.4 mV (Na_v_1.6) and 9.5 mV (Na_v_1.3) with increasing temperature from 15°C to 35°C (Fig. 2, A and C; and Fig. S2). These shifts were significant in both temperature increments for Na_v_1.3 and Na_v_1.7, while for Na_v_1.5 and Na_v_1.6, only the temperature rise from 15°C to 25°C produced a significant shift. Except for Na_v_1.6 at 35°C, we also observed a steepening of the conductance curves and decreased slope factors *k* with increasing temperature (Fig. 2 and Table 1).

**Table 1.** Summary of electrophysiological parameters determined for Na_v_1.3, Na_v_1.5, Na_v_1.6, and Na_v_1.7

	Na_v_1.3			Na_v_1.5			Na_v_1.6			Nav1.7		
	15°C	25°C	35°C	15°C	25°C	35°C	15°C	25°C	35°C	15°C	25°C	35°C
**Activation parameter**												
*V*_*1/2*_ * (mV)*	−18.8 ± 0.5	−25.0 ± 0.6	−28.3 ± 0.6	−42.5 ± 0.7	−48.9 ± 0.4	−51.2 ± 0.6	−28.9 ± 0.6	−33.5 ± 0.5	−34.3 ± 0.8	−27.5 ± 0.7	−32.0 ± 0.5	−- 35.4 ± 0.5
*k*	8.2 ± 0.1	6.0 0.2	4.7 ± 0.2	6.6 ± 0.2	4.7 ± 0.2	4.5 ± 0.2	5.3 ± 0.2	3.9 ± 0.2	4.6 ± 0.2	6.3 ± 0.2	4.5 ± 0.2	4.2 ± 0.2
*Time-to-peak (ms) (0 mV)*	1.36 ± 0.05	0.53 ± 0.02	n.a.	0.78 ± 0.02	0.47 ± 0.01	n.a.	0.68 ± 0.01	0.45 ± 0.01	n.a.	0.64 ± 0.01	0.38 ± 0.005	n.a.
*n*	90	84	64	80	77	50	71	36	38	94	80	85
**Inactivation parameter**												
*V*_*1/2*_ *(mV)*	−68.6 ± 0.5	−69.5 ± 0.7	−67.4 ± 0.6	−82.7 ± 0.6	−82.4 ± 0.4	−84.1 ± 0.8	−65.9 ± 0.6	−62.0 ± 0.5	−64.2 ± 0.8	−76.2 ± 0,9	−75.4 ± 0.5	−76.1 ± 0.7
*k*	−11.5 ± 0.2	−9.2 ± 0.1	−8.0 ± 0.1	−8.3 ± 0.1	−6.1 ± 0.1	−5.6 ± 0.1	−7.55 ± 0.1	−6.0 ± 0.1	−6.3 ± 0.1	−10.3 ± 0.3	−7.0 ± 0.1	−7.3 ± 0.1
*n*	93	102	99	94	128	96	71	78	74	138	135	121
**Inactivation kinetics**												
τ *(ms) (0 mV)*	4.80	1.20	0.54	1.72	0.70	0.42	1.49	0.69	0.38	1.13	0.53	0.28
*n*	90	78	59	80	76	48	71	35	36	93	76	79
*Max I*_*pers*_ *(% from I*_*max*_*)*	21.0 ± 0.6	5.2 ± 0.2	1.1 ± 0.1	6.4 ± 0.3	2.0 ± 0.1	0.9 ± 0.1	3.6 ± 0.2	1.2 ± 0.1	n.a.	2.5 ± 0.3	0.7 ± 0.1	n.a.
*n*	90	79	64	80	77	50	71	36	n.a.	94	76	81
**Recovery from fast inactivation**												
*%* τ *fast*	76.3	69.9	37.1	71.0	70.1	62.3	81	75.3	39.5	87.0	81.4	51.0
τ *fast (ms)*	19.7	7.6	3.5	93.1	34.0	23.5	10.2	2.7	2.7	49.2	13.3	8.5
τ *slow (ms)*	250.9	285.5	193.4	570.0	287.4	246.8	398.6	303.9	252.3	447.8	428.8	621.9
*n*	58	75	64	48	101	66	47	70	25	74	87	61

n.a., not available.

We analyzed the time to peak and the time from the onset of the voltage step until the maximum inward sodium current is reached. The channel opening was between 1.5 times (Na_v_1.6) and 2.6 times (Na_v_1.3) faster at 25°C compared with 15°C. At 35°C, the activation became too fast to be accurately dissolved and no reliable analysis was possible. Taken together, the hyperpolarized *V*_*1/2*_ as well as the accelerated opening suggests a strong impact of temperature on the overall excitability of Na_v_s.

### Effects of temperature on the inactivation properties of Na_v_ subtypes

Na_v_s inactivate quickly within milliseconds upon activation. To assess the channels’ steady-state fast inactivation, we used the voltage protocol shown in Fig. 2. No significant shifts in the voltage dependence of fast inactivation caused by temperature variation were observed, except for Na_v_1.6, whose *V*_*1/2*_ at 25°C was 3.9 mV more depolarized compared to 15°C (P = 0.0052 in one-way ANOVA with Sidak’s multiple comparisons test; Fig. 2, B and D; Fig. S2; and Table 1). Steady-state fast inactivation was complete in all tested channels except for Na_v_1.3 at 15°C. Similar to the activation process, the IV curves became less steep at 15°C, as indicated by increased slope factors (Table 1).

**Figure 3.**
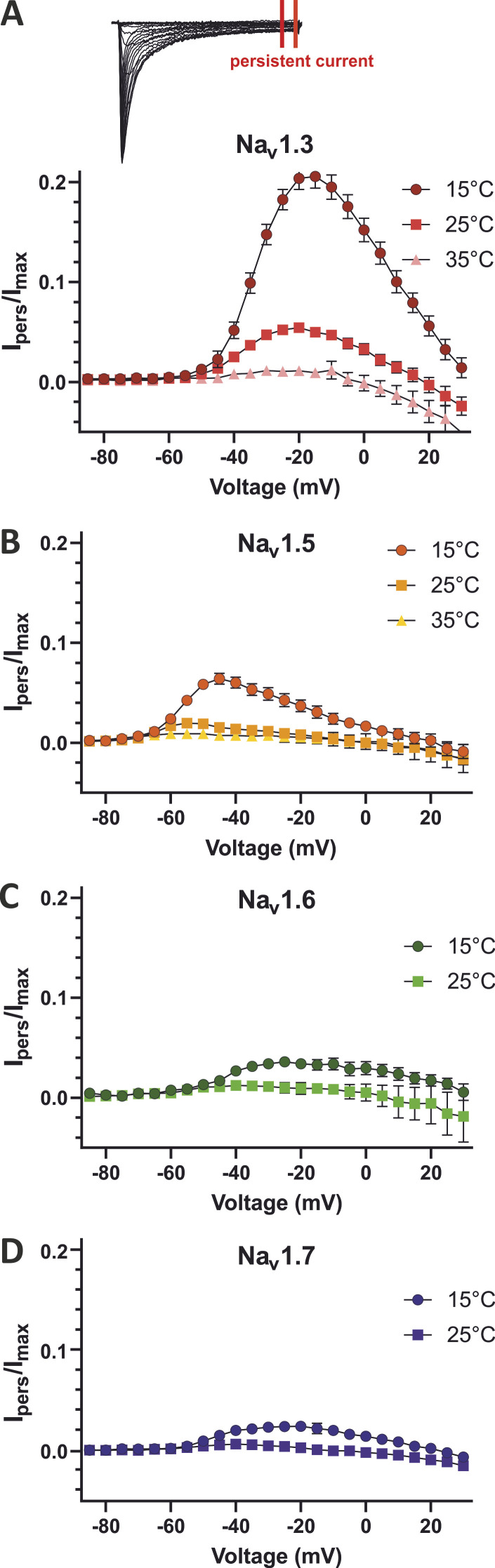
**Temperature strongly modulates the inactivation kinetics. (A–D)** Persistent sodium current *I*_*pers*_ normalized to the maximum inward current *I*_*max*_ for (A) Na_v_1.3, (B) Na_v_1.5, (C) Na_v_1.6, and (D) Na_v_1.7 at 15°C, 25°C, and 35°C. All values are shown as mean ± 95% confidence interval.

The persistent current was enhanced with lowered temperature. This was the case for all investigated subtypes (Fig. 3, A–D), but it was especially prominent for Na_v_1.3, where the maximum persistent current *I*_*pers*_ (as percentage of the maximum inward current) was about four times higher at 15°C (21.0% ± 0.6%) compared to 25°C (5.2% ± 0.2%), while at 35°C nearly no persistent current was detectable (1.1% ± 0.1%; Fig. 3 A). These results indicate that the voltage dependence of fast inactivation is only slightly affected, but the inactivation kinetics are strongly modulated by temperature.

**Figure 4.**
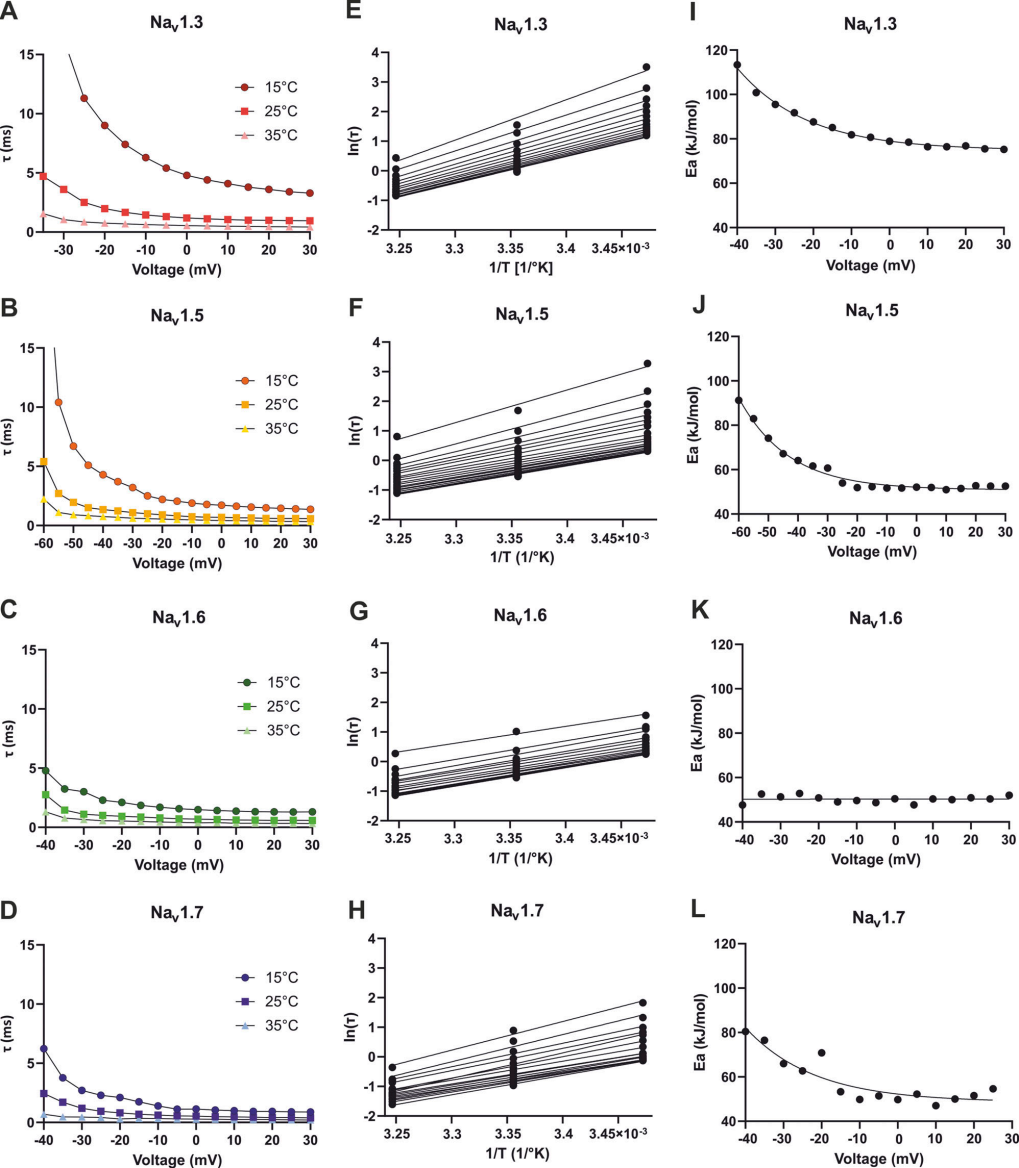
**Kinetic and thermodynamic analysis of fast inactivation. (A–D)** Inactivation time constant τ extracted from mono-exponential fits to the averaged traces at 15°C, 25°C, and 35°C. **(E–H)** Arrhenius plots calculated from inactivation time constant τ to determine the activation energy for the inactivation process at each voltage. **(I–L)** Summary of the Arrhenius analysis. Activation energy (E_a_) for inactivation plotted as a function of voltage. E_a_ was determined following the Arrhenius equation: $\alpha =e^{-z\left(\alpha \right)V}\times e^{-\frac{E_{a}\left(\alpha \right)}{RT}}$ (compare methods).

We determined the inactivation time constant τ by a single exponential fit to further quantify the effect of temperature on inactivation kinetics. Rising temperature led to an acceleration of the inactivation kinetics with declining τ-values. Compared to 25°C, τ was ∼2.1–2.5 times larger at 15°C and ∼1.7–1.9 times smaller at 35°C for Na_v_1.5, Na_v_1.6, and Na_v_1.7 at the voltage step to 0 mV (Table 1; and Fig. 4, D, G, and J). For Na_v_1.3, lowering the temperature from 35°C to 25°C led to a 2.2-fold and further cooling from 25°C to 15°C led to a fourfold increase of τ (Fig. 4 A). Compared with the τ*-*value of Na_v_1.7 at 15°C (1.13 ms) that of Na_v_1.3 was 4.2 times larger (4.80 ms). This as well as the enhanced persistent current may indicate a special sensitivity of this channel’s inactivation kinetics toward cooling.

An Arrhenius analysis was performed for potentials less negative than −40 mV (and −60 mV for Na_v_1.5). It revealed roughly flat plots only for Na_v_1.6, with an estimated *E*_*a*_ of ≈50 kJ/mol (Fig. 4, G and K). Because τ = 1/(α + β), this indicates that the forward rate constant α and the backward rate constant β from the open to the inactivated state have a similar *E*_*a*_, and τ ≈ 1/α can be assumed. For the other subtypes, *E*_*a*_ seems to depend on voltage, reflected in the curved Arrhenius plots (Fig. 4, E, F, and H) as well as the not linear progression of *E*_*a*_ plotted against voltage (Fig. 4, I, J, and L). The curves became flat only for potentials more positive than −10 mV for Na_v_1.3 and Na_v_1.7 and −20 mV for Na_v_1.5, thus τ ≈ 1/α can be assumed only for this voltage range.

The averaged E_a_(α) for potentials less negative than −10 mV was ≈52 kJ/mol for Na_v_1.5 and ≈51 kJ/mol for Na_v_1.7. For Na_v_1.3, it was 1.5 times larger with ≈79 kJ/mol. This indicates that for Na_v_1.3 more energy is needed to achieve fast inactivation, which may explain the strongly slowed inactivation of this subtype at 15°C.

### The recovery from inactivation displays a high dependency on temperature

**Figure 5.**
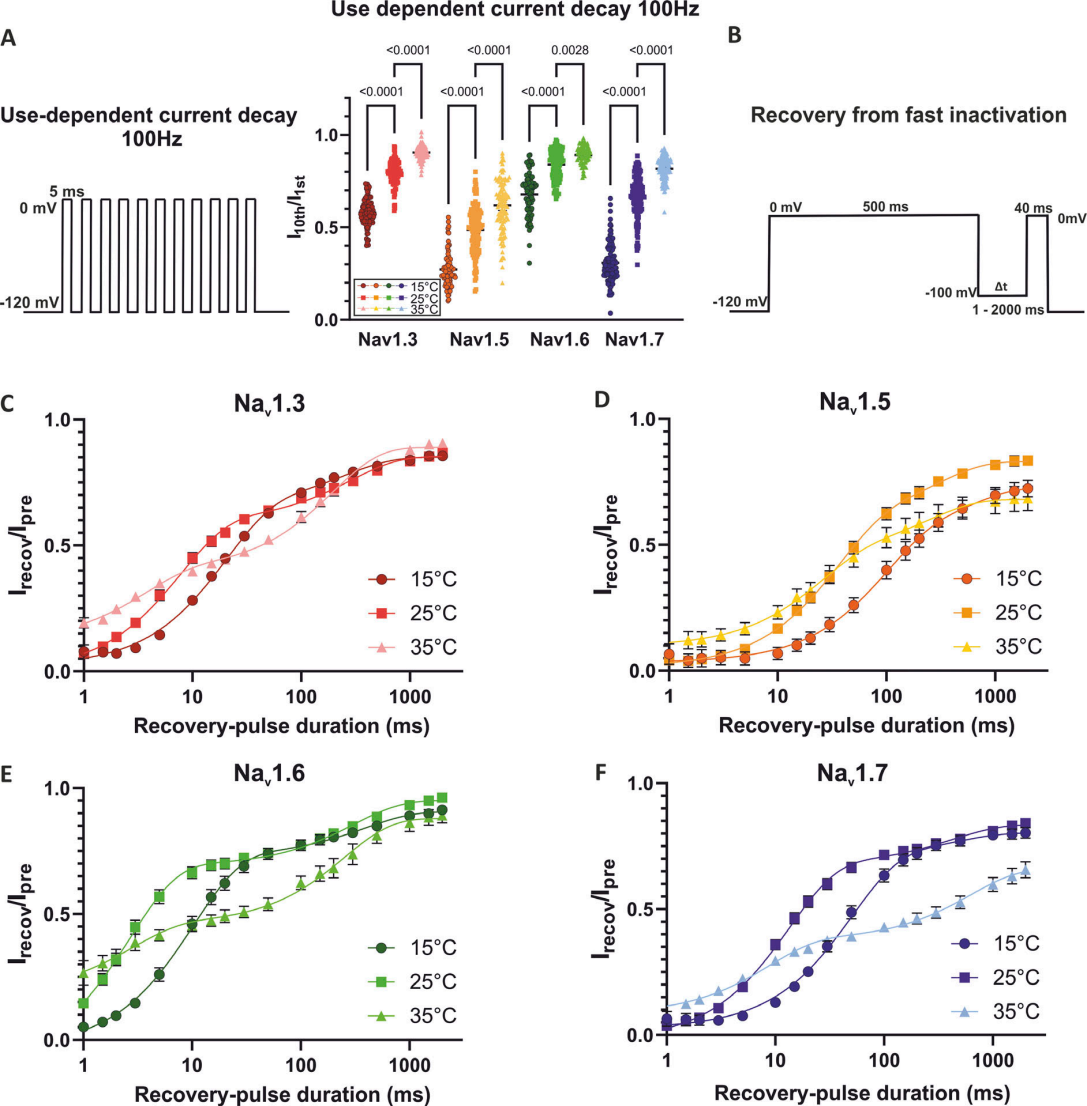
**Temperature effects on use-dependent current decay and recovery from fast inactivation. (A)** Use-dependent current decay of Na_v_1.3, Na_v_1.5, Na_v_1.6, and Na_v_1.7 at 15°C, 25°C, and 35°C at 100 Hz represented as the normalized current amplitude of the 10th to the 1st inward current. Voltage protocol shown on the left. All channel subtypes show a statistically significant use-dependent current decay with decreasing temperature. **(B)** Voltage protocol used to determine recovery from fast inactivation. **(C–F)** Recovery from fast inactivation at different temperatures. Normalized current amplitude as a function of recovery-pulse duration, voltage protocol shown on the top right. Data are shown as mean ± 95% confidence interval. One-way ANOVA with Sidak’s multiple comparisons test.

Because it is an important determinant of channel availability in high-frequency firing neurons, we investigated the channel’s use-dependent current decay using the voltage protocol shown in Fig. 5 A. Comparing the current of the first to the current of the last peak in a series of 10 5-ms depolarizations at 100 Hz, we observed a significantly stronger use-dependent current decay at 15°C compared to 25°C and 35°C for all investigated subtypes (Fig. 5 A).

As recovery from fast inactivation has a large impact on the use-dependent current decline, we used the voltage protocol shown in Fig. 5 B to measure this gating characteristic. With warming from 15°C to 25°C, we observed in general an acceleration of the channel’s recovery, expressed in the left shift of the curves (Fig. 5, C–F) as well as decreased τ_*fast*_ values (Table 1). At 35°C, lower τ_*fast*_ values indicate an even faster recovery compared to the one at 25°C, but the proportion of fast recovering channels, *% *τ_*fast*_, decreased drastically, resulting in a flattening of the recovery curves and a much higher impact of the slow recovering process, represented by τ_*slow*_. With a prepulse duration of 500 ms, as used in this protocol, it is possible that a part of the channels is already slow inactivated. Our results suggest that, while the recovery from fast inactivation is accelerated at near physiological temperature, the onset of slow inactivation is enhanced at the same time.

### Temperature-induced changes in the excitability of the IEM-mutation Na_v_1.7/L823R

**Figure 6.**
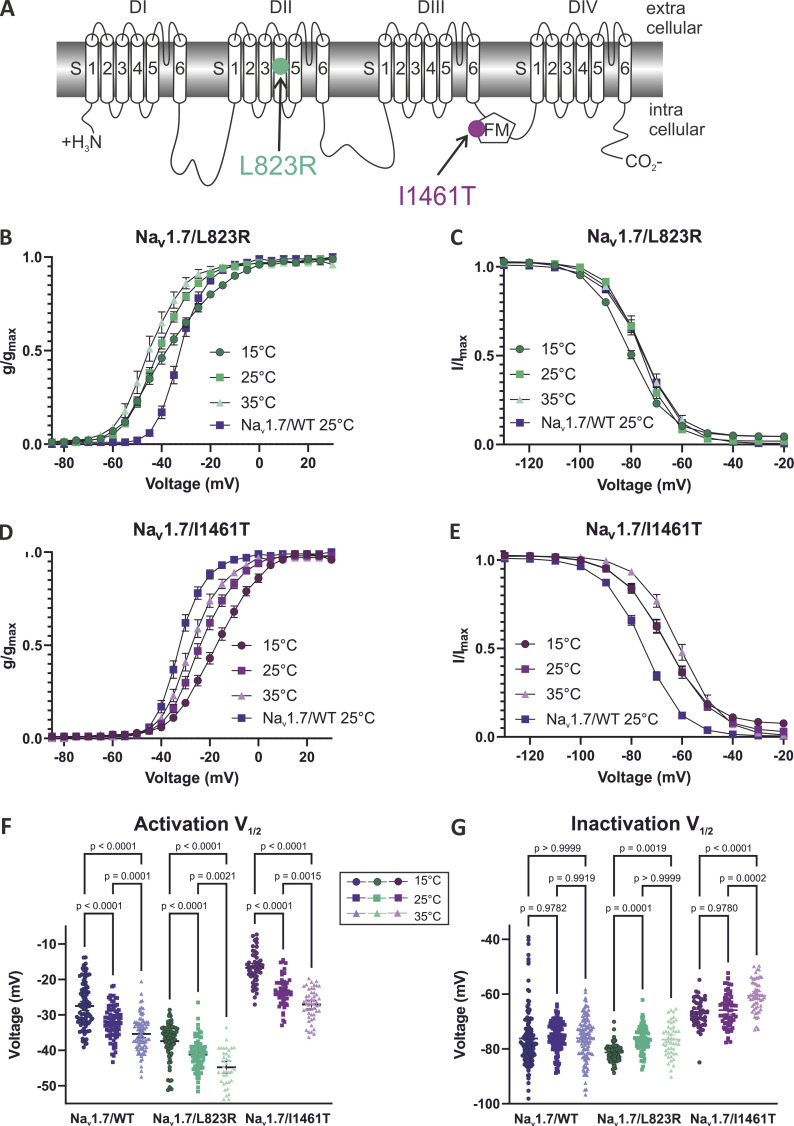
**Voltage dependence of activation and steady-state fast inactivation of the IEM mutation Na**_**v**_**1.7/L823R and the PEPD mutation Na**_**v**_**1.7/I1461T compared with Na**_**v**_**1.7/I1461TWT. (A)** 2-D scheme of the Na_v_1.7 channel showing the location of the affected amino acids for the mutation L823R (green symbol) and the mutation I1461T (purple symbol). **(B)** The G-V dependence of Na_v_1.7/L823R is shifted to more hyperpolarized potentials compared with WT. **(C)** Steady-state fast inactivation of L823R is slightly shifted to more negative potentials at 15°C. **(D and E)** Activation (D) and steady-state (E) fast inactivation are shifted to more depolarized potentials for Na_v_1.7/I1461T. **(F and G)** Values of half-maximal voltage dependence of activation (F) and inactivation (G) obtained from Boltzmann fit for individual traces. Data are shown as mean ± 95% confidence interval. One-way ANOVA with Sidak’s multiple comparisons test. Exact P values are indicated.

We showed that temperature has profound effects on Na_v_ gating of WT channels. Here, we investigated how changes in temperature affect the biophysics of the disease-related IEM-causing mutation Na_v_1.7/L823R. The *V*_*1/2*_ of Na_v_1.7/L823R was significantly shifted to more hyperpolarized potentials with increasing temperature (Fig. 6 F and Table 2). Compared with Na_v_1.7/WT, the mutation exhibited an ∼10 mV hyperpolarizing shift of activation *V*_*1/2*_ at all temperatures tested (P < 0.0001 in a Student’s *t* test; Fig. 6, B and F; and Table 2). This represents a fitting explanation for the patient’s phenotype with increased temperature triggering pain attacks. At 15°C, the *V*_*1/2*_ of the mutation (*V*_*1/2*_ = −37.4 ± 0.6 mV) was close to the *V*_*1/2*_ of the WT at 35°C (*V*_*1/2*_ = −35.4 ± 0.5 mV), suggesting that the voltage dependence of the channel’s activation is closer to the physiological (WT) conditions at colder temperature. Additionally, the L823R mutation renders the conduction curves less steep, with a slope factor *k* of 10.6 ± 0.3 mV at 15°C, compared with the slope factor of 6.3 ± 0.2 mV for WT at 15°C (Table 2). Comparing the time to peak values of WT and L823R mutation, we observed a slower activation at 25°C (P < 0.005 in multiple unpaired *t* tests), which was not detectable at 15°C.

**Table 2.** Summary of electrophysiological parameters determined for Na_v_1.7 and the two mutations Na_v_1.7/L823R and Na_v_1.7/I1461T

	Na_v_1.7/WT			Na_v_1.7/L823R			Na_v_1.7/I1461T		
	15°C	25°C	35°C	15°C	25°C	35°C	15°C	25°C	35°C
**Activation parameter**									
*V*_*1/2*_ *(mV)*	−27.5 ± 0.7	−32.0 ± 0.5	−35.4 ± 0.5	−37.4 ± 0.6	−41.2 ± 0.5	−44.8 ± 0.8	−16.7 ± 0.7	−23.4 ± 0.6	−27.1 ± 0.6
*k*	6.3 ± 0.2	4.5 ± 0.2	4.2 ± 0.2	10.6 ± 0.3	7.0 ± 0.2	6.3 ± 0.3	8.5 ± 0.2	7.0 ± 0.2	6.0 ± 0.2
*Time-to-peak (ms)*	0.64 ± 0.01	0.38 ± 0.005	n.a.	0.71 ± 0.05	0.43 ± 0.01	n.a.	1.07 ± 0.06	0.47 ± 0.01	n.a.
*n*	94	80	81	73	88	37	50	50	51
**Inactivation parameter**									
*V*_*1/2*_ *(mV)*	−76.2 ± 0,9	−75.4 ± 0.5	−76.1 ± 0.7	−81.3 ± 0.4	−76.4 ± 0.5	−76.6 ± 0.9	−67.1 ± 0.7	−65.8 ± 0.8	−60.6 ± 0.8
*k*	−10.3 ± 0.3	−7.0 ± 0.1	−7.3 ± 0.1	−6.5 ± 0.2	−5.1 ± 0.1	−5.8 ± 0.2	−7.8 ± 0.2	−8.1 ± 0.2	−6.9 ± 0.2
*n*	138	135	121	70	81	49	49	66	57
**Inactivation kinetics**									
τ *(ms) (0 mV)*	1.13	0.53	0.28	1.47	0.60	0.35	2.73	0.70	0.33
*n*	93	76	79	71	84	29	50	47	42
*Max I*_*pers*_ *(% from I*_*max*_*)*	2.5 ± 0.3	0.7 ± 0.1	n.a.	10.0 ± 0.4	3.8 ± 0.1	2.4 ± 0.4	14.4 ± 1.4	3.9 ± 0.6	1.8 ± 0.2
*n*	94	76	81	73	84	37	50	48	51
**Recovery from fast inactivation**									
*%* τ *fast*	87.0	81.4	51.0	86.4	87.3	56.9	78.4	51.3	31.7
τ *fast (ms)*	49.2	13.3	8.5	89.0	20.0	11.0	22.4	8.34	589.2
τ *slow (ms)*	447.8	428.8	621.9	403.4	315.4	550.1	337.3	523.5	589.2
*n*	74	87	61	50	62	24	35	54	45

n.a., not available.

In our experiments, we did not see any significant shift in the steady-state fast inactivation *V*_*1/2*_ of the mutation vs. WT at 35°C or 25°C (P = 0.6953 at 35°C, P = 0.1452 at 25°C in a Student’s *t* test), but at 15°C steady-state, fast inactivation, *V*_*1/2*_, was shifted by 4.8 mV to more hyperpolarized potentials for the mutation (P < 0.0001 in a Mann-Whitney test; Fig. 6, C and G; and Table 2). This left shift in steady-state inactivation would also render neurons less excitable at 15°C.

**Figure 7.**
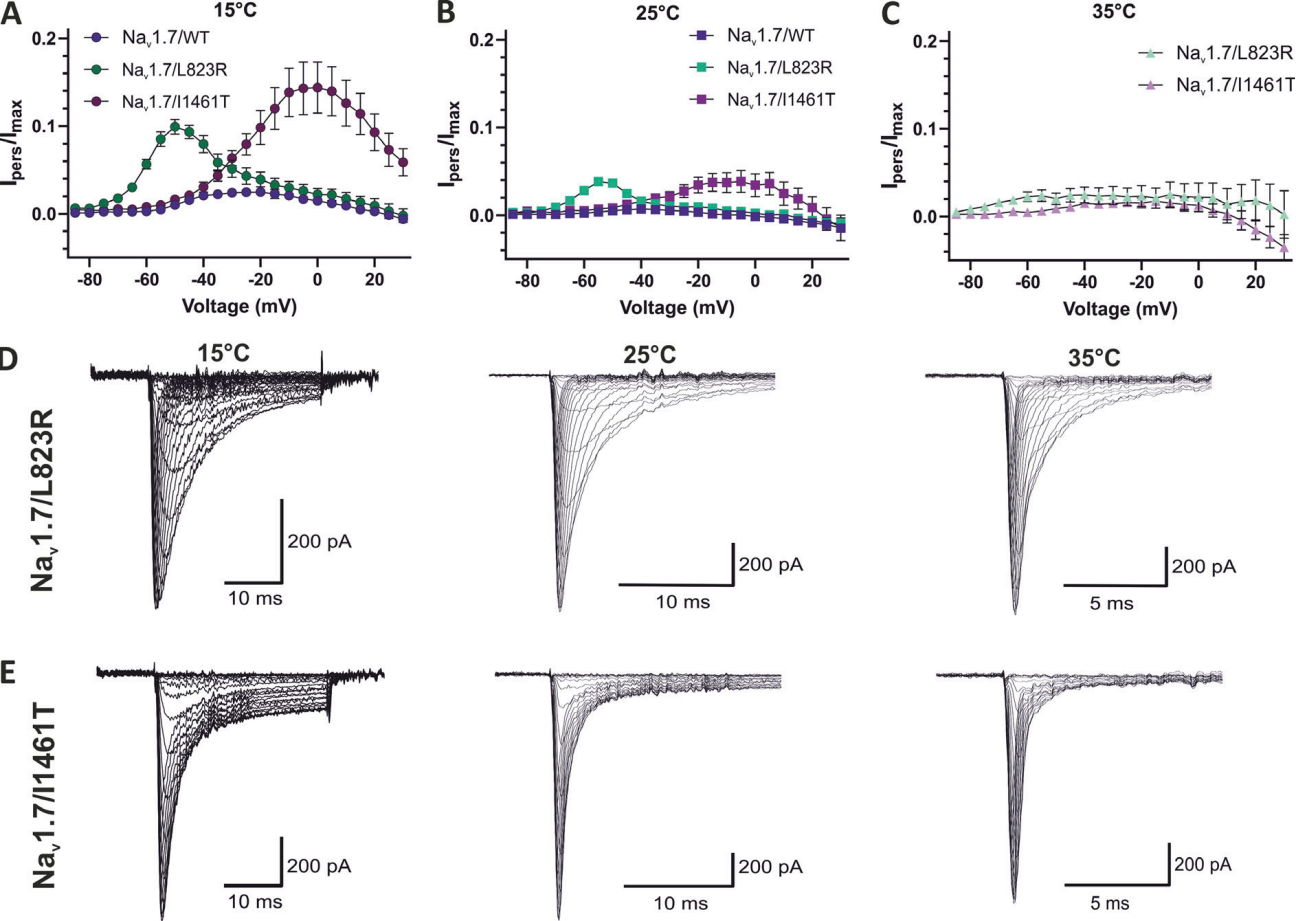
**Cold temperature has a stronger impact on both mutation’s inactivation kinetics than on Na**_**v**_**1.7 WT. (A–C)** Persistent current (I_pers_) normalized to the maximal inward current (I_max_). At (A) 15°C and (B) 25°C, the persistent current was strongly enhanced for both mutations compared with WT. Data are shown as mean ± 95% confidence interval. **(D and E)** Average of all included current traces for (D) Na_v_1.7/L823R and (E) Na_v_1.7/I1461T.

We noticed a 4- and 5.4-fold larger persistent current for the mutation at 15°C and 25°C, respectively (10.0% ± 0.4% at 15°C, 3.8% ± 0.1% at 25°C), than for the WT (2.5% ± 0.3% at 15°C, 0.7% ± 0.1% at 25°C; Fig. 7, A and B; and Table 2). Increased leak current made accurate analysis of the persistent current difficult at 35°C.

**Figure 8.**
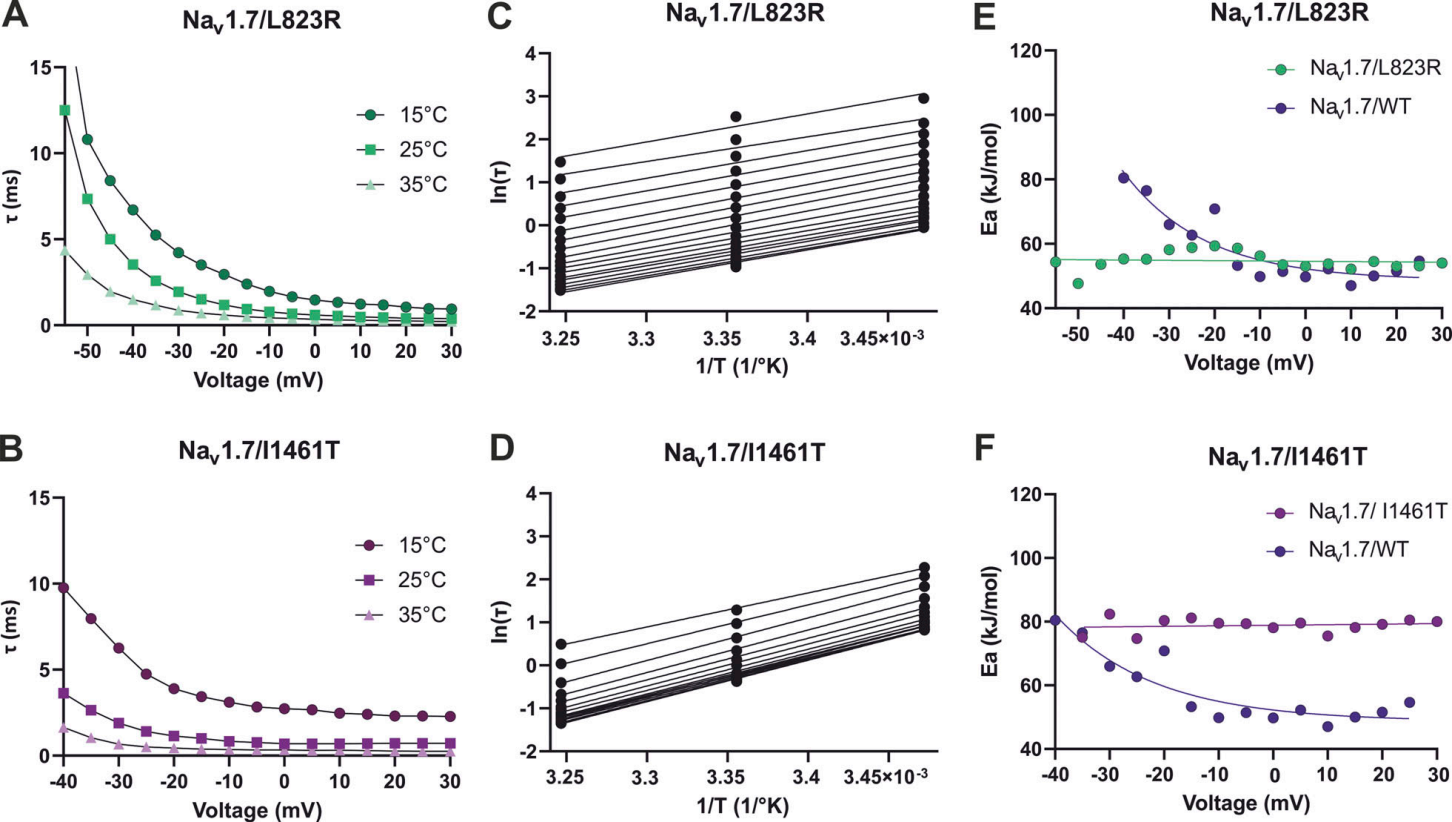
**Kinetic and thermodynamic analysis of fast inactivation in Na**_**v**_**1.7 mutations. (A and B)** Inactivation time constant τ at 15°C, 25°C, and 35°C for (A) Na_v_1.7/L823R and (B) Na_v_1.7/I1461T. **(C and D)** Arrhenius plots calculated from τ to determine *E*_*a*_ for the inactivation process at each voltage. **(E and F)** Summery of the Arrhenius analysis with *E*_*a*_ of the inactivation process plotted as a function of voltage, comparing Na_v_1.7 WT with (E) Na_v_1.7/L823R and (F) Na_v_1.7/I1461T. The average of all included current traces was used for this analysis.

The inactivation time constant *τ* of Na_v_1.7/L823R at 0 mV was ∼1.2 times larger than for WT (Table 2 and Fig. 8 A). Thermodynamic analysis revealed Arrhenius plots (Fig. 8 B) that were roughly parallel for the mutation for the voltage range above −55 mV. With the average slope of this curve, *E*_*a*_ was estimated to be ≈55 kJ/mol, which is quite close to *E*_*a*_ ≈ 51 kJ/mol for Na_v_1.7/WT. In contrast to WT, no voltage dependence of *E*_*a*_ was observed (Fig. 8 C).

**Figure 9.**
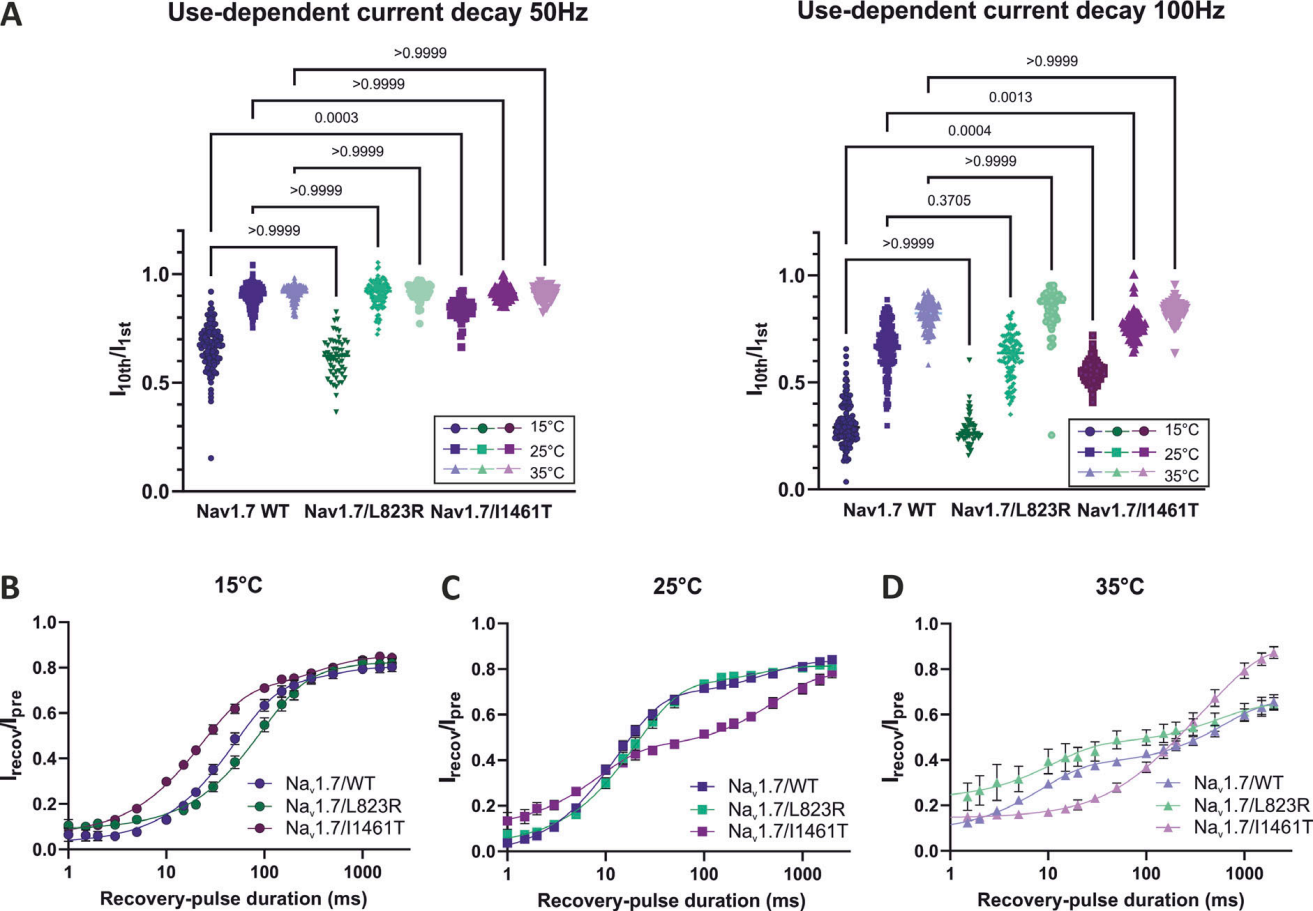
**Na**_**v**_**1.7/I1461T shows a significantly different behavior in use-dependent inactivation and recovery from inactivation compared to Na**_**v**_**1.7/WT and Na**_**v**_**1.7/L823R. (A)** Use-dependent current decay of Na_v_1.7/WT, Na_v_1.7/L823R, and Na_v_1.7/I1461T at 15°C, 25°C, and 35°C represented as the normalized current amplitude of the 10th to the 1st inward current. At 15°C, Na_v_1.7/I1461T displayed significantly smaller use-dependent inactivation compared with WT. **(B–D)** Recovery from fast inactivation, normalized current amplitude as a function of recovery-pulse duration. Data are shown as mean ± 95% confidence interval. Kruskal–Wallis test with Dunn’s multiple comparisons test. Exact P values are indicated. For recovery time-constants, see Table 2.

Regarding the use-dependent current decay, we did neither observe any significant difference between L823R and WT for 50 Hz nor for 100 Hz at 15°C, 25°C, and 35°C (Fig. 9 A). Testing for the recovery from fast inactivation, at 15°C, the recovering curve of the WT was slightly shifted toward shorter recovery times compared with the mutation (Fig. 9 B), indicating a slower recovery process for the mutated channel. This was also reflected in the τ_*fast*_ value (45.63 ms for WT, 88.41 ms for L823R). At 25°C, the recovery curves were almost overlapping with similar τ_*fast*_ values (Table 2 and Fig. 9 C), and at 35°C, the L823R channel recovered faster than the WT channel (Fig. 9 C), but this result has to be treated with caution because the measurement of the mutation became unstable at 35°C.

**Figure 10.**
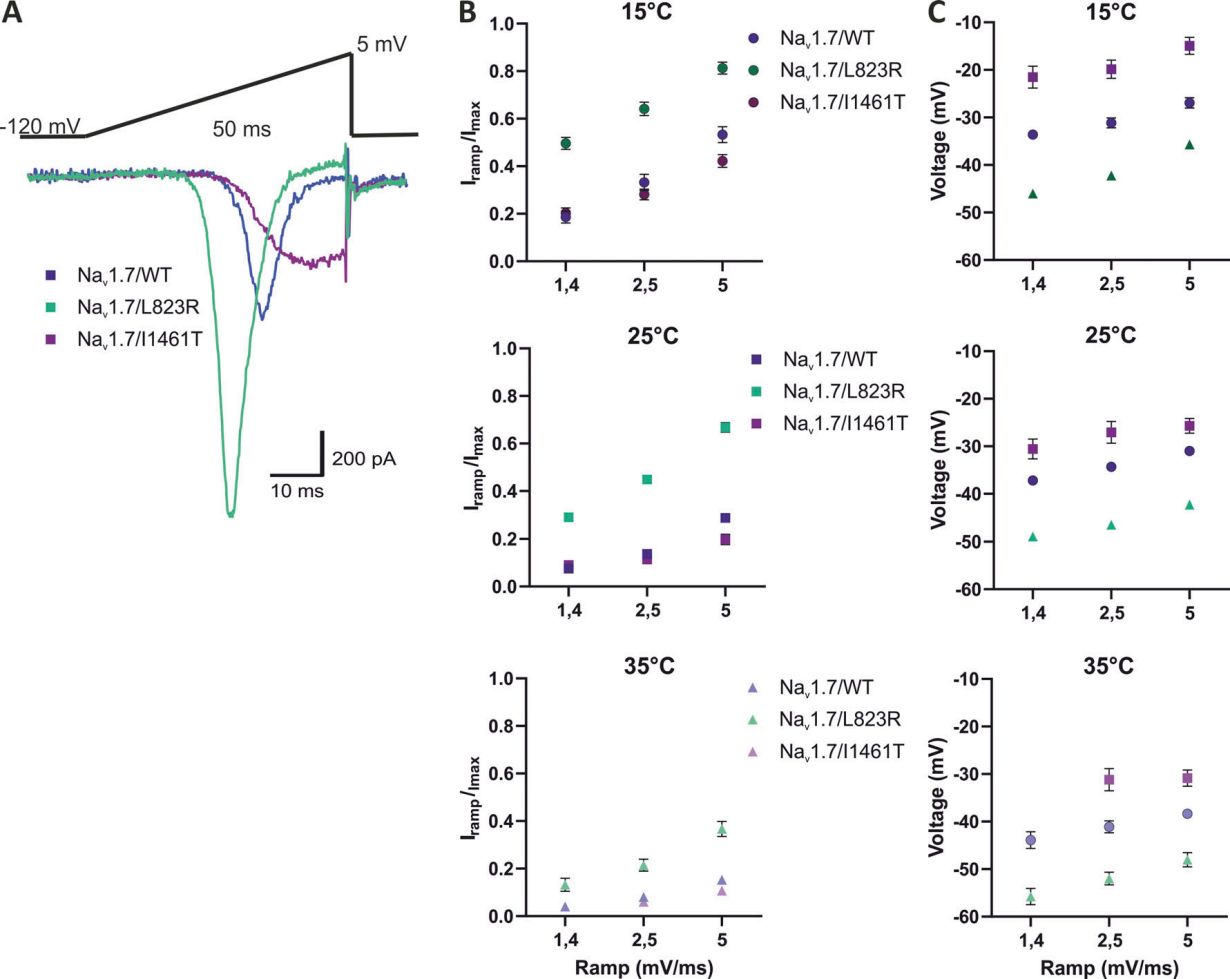
**Na**_**v**_**1.7/L823R shows strongly enhanced ramp currents over the whole temperature range. (A)** Example ramp-current elicited by 2.5 mV/ms ramps at 25°C. Comparison of Na_v_1.7WT (blue), Na_v_1.7/L823R (green), and Na_v_1.7/I1461T (purple). **(B)** Ramp-current normalized to maximal inward current and plotted against ramp speed. The current increased with cooling and enhanced ramp speed. Ramp current of Na_v_1.7/L823R was clearly stronger than the one of Na_v_1.7/WT and Na_v_1.7/I1461T. **(C)** Voltage at which the peak ramp current occurs. With decreasing temperature or increasing ramp speed it is shifted to more depolarized potentials. Data are shown as mean ± 95% confidence interval.

It was described before that other mutations in Na_v_1.7 causing IEM increase ramp currents evoked by the application of slow depolarizing pulses (Han et al., 2007). Here, we applied three different ramps from a holding potential of −120 mV with a speed of 1.4, 2.5, and 5 mV/ms. Fig. 10 A shows example traces of ramp currents elicited by a 2.5 mV/ms ramp at 25°C for Na_v_1.7/WT and both mutations. The ratio peak ramp current *I*_*ramp*_ to the maximum inward current *I*_*max*_ increased with decreasing temperature as well as with increasing ramp speed for all investigated subtypes (Fig. 10). Comparing Na_v_1.7/L823R with Na_v_1.7/WT, we observed a significant increase in ramp currents of the mutation at all tested temperatures (Fig. 10 B; P < 0.0001 in Student’s *t* tests). Furthermore, the voltage at which the peak ramp current occurs was shifted by 9–12 mV to more hyperpolarized potentials for the mutation compared with WT. Comparing 35°C and 15°C, we observed an ∼10 mV shift to more depolarized potentials when lowering the temperature to 15°C. These results may indicate higher sensitivity of the channels to slow subthreshold stimuli at temperatures between 15°C and 35°C.

### Cooling induces an enhanced impaired inactivation for the PEPD mutation Na_v_1.7/I1461T

The PEPD mutation Na_v_1.7/I1461T, affecting the highly conserved inactivation motif in the DIII/IV-linker (Fig. 6 A), was described before to induce changes in fast as well as in slow inactivation (Fertleman et al., 2006; Jarecki et al., 2008; Sheets et al., 2011). Here, we observed a shift to more depolarized potentials of steady-state fast inactivation compared with WT (Fig. 6, E and G). The *V*_*1/2*_ was ∼10 mV less negative for the mutation at 15°C and 25°C (P < 0.0001 in Student’s *t* tests; Table 2). Elevating the temperature to 35°C shifted the IV-curve to even more depolarized potentials, an effect we did not observe in the steady-state fast inactivation of WT channels (Fig. 6 G and Table 2).

For activation *V*_*1/2*_, we also observed shifts of 8.3 mV (35°C), 8.6 mV (25°C), and 11.8 mV (15°C) to more depolarized potentials compared with WT (Fig. 6 E and Table 2). Regarding the temperature dependence of activation, the mutation showed the same effect as WT with significant shifts in *V*_*1/2*_ to more hyperpolarized potentials, from −16.7 ± 0.7 mV at 15°C to −27.1 ± 0.6 mV at 35°C (Fig. 6 F). We observed a strong increase in persistent current for the mutation with a normalized persistent current *I*_*pers*_ of 14.4% ± 1.4% at 15°C for Na_v_1.7/I1461T and only 2.5% ± 0.3% for WT (Fig. 7, A and B). Due to the increasing leak current at 35°C, the calculation of the persistent current was not precise at depolarized potentials (Fig. 7, C and E).

The inactivation kinetics were slowed with decreasing temperature (Fig. 8 B and Table 2). τ*-*values were exemplary for the voltage step to 0 mV, 1.2 and 1.3 times larger at 35°C and 25°C compared with WT, but 1.9 times larger at 15°C. Furthermore, *E*_*a*_ could be estimated for Na_v_1.7/I1461T for potentials less negative than −40 mV and showed no dependence on voltage in this range (Fig. 8 F). Averaged *E*_*a*_ of fast inactivation was ≈78 kJ/mol, a 1.6-fold increase compared with WT. The increased activation energy for fast inactivation may be explained by the impaired inactivation process of the mutation and causes probably the observed persistent current as well as the slowed inactivation.

Testing the channel’s use-dependent current decay at 50 and 100 Hz, Na_v_1.7/I1461T displayed a stronger current decay with decreasing temperature, similar to Na_v_1.7/WT. However, at 15°C it was significantly smaller compared with WT for 50 Hz and 100 Hz (Fig. 9 A).

A faster recovery from fast inactivation for the I1461T mutation was described before, which provides a possible explanation for this effect. Investigating the recovery from fast inactivation with a 500 ms prepulse to 0 mV revealed a speeding of the mutation’s recovery compared with WT at 15°C, with τ_*fast*_ of 22.4 ms for I1461T and 45.6 ms for WT (Fig. 9 B and Table 2). Interestingly, at 25°C, the recovery behavior of the mutant was faster, as observed for the WT channel, but in addition the proportion of fast recovering channels (% τ_*fast*_) decreased from 78.4% at 15°C to 51.3% at 25°C (Table 2). Thus, the proportion of channels that recover slowly becomes more dominant. This resulted in a flattening of the curve (Fig. 9 C) at 25°C and 35°C, which was only observed at 35°C for the WT (Fig. 5 F). Even though τ_*fast*_ is still smaller and for recovery-pulse durations up to 10 ms a higher proportion of mutant channels is recovering compared with WT, for longer recovery periods between 20 and 2,000 ms the fraction of recovered channels is reduced for the mutation (Fig. 9 C). This may indicate that slow inactivation is already occurring during the 500 ms prepulse at 25°C. At 35°C, the majority of the channels seem to mainly recover slowly, with a % τ_*fast*_ of only 31.7% and a substantial proportion of Nav1.7/I1461T channels that just starts recovering when repolarized for periods longer than 50 ms (Fig. 9 D).

Ramp currents of Na_v_1.7/I1461T were not significantly changed compared with WT at 15°C, 25°C, and 35°C for 1.4 and 2.5 mV/ms ramps, while 5 mV/ms ramps were significantly smaller for the mutation (P < 0.0001 in a Student’s *t* test; Fig. 10 B). The voltage at which peak ramp current occurs was, compared with WT, shifted by 5–10 mV to more depolarized potentials (Fig. 10 C)_._

## Discussion

In this study, we investigated the effects of temperature on four different Na_v_ subtypes and two mutations of Na_v_1.7, which are linked to the inherited pain syndromes IEM and PEPD, under standardized conditions at 15°C, 25°C, and 35°C. We reveal a pronounced sensitivity of Na_v_1.3 fast inactivation kinetics to lowered temperature, resulting in a striking persistent current that may play a role in injury-induced cold allodynia. Biophysical effects of pain-linked mutations in Na_v_1.7 were enhanced by warmth in IEM and by cooling in PEPD, which may explain the clinically observed specific triggers of these diseases.

### Temperature-induced enhancement of Na_v_ activation

Here, we systematically investigated the temperature dependence of activation of Na_v_1.3, Na_v_1.5, Na_v_1.6, and Na_v_1.7, and observed a hyperpolarizing shift in the voltage dependence of activation with increasing temperature (Figs. 2 and 6). Due to the nature of our experimental setting, we can directly compare temperature effects on the channels, and a large number of experiments due to the high throughput patch clamp further increases the quality of our data. Temperature effects on Na_v_ activation were investigated before, mostly using a manual patch clamp (Sarria et al., 2012; Thomas et al., 2009; Almog et al., 2022; Egri et al., 2012). However, reports on the direction of heat-induced shifts in the voltage dependence of Na_v_ activation vary in literature depending on the experimental conditions, and some studies show no significant shift (Touska et al., 2018; Rosen, 2001; Ye et al., 2018) or even a shift of *V*_*1/2*_ to more depolarized potentials (Zimmermann et al., 2007) with increasing temperature. In our study, we used comparable conditions for all temperatures investigated, and due to the high-throughput setting, our experiments have sufficient *n* to support our findings. The voltage dependence of Na_v_ activation is an important determinant for the excitability of the tissue it is expressed in. Thus, warmth-induced shifts to more hyperpolarized potentials may explain for example increased neuronal excitability in febrile seizures (Thomas et al., 2009; Ye et al., 2018) or fever-triggered arrhythmic events in normal hearts (Pasquie et al., 2004).

### *V*_*1/2*_ of steady-state fast inactivation is only slightly affected by temperature for WT Na_v_s

In this study, Na_v_1.3, Na_v_1.5, and Na_v_1.7 exhibited no significant shifts in the midpoint of fast inactivation induced by temperature, and in the case of Na_v_1.6, we observed only a slight shift to more hyperpolarized potentials comparing 15–25°C (Fig. 3). For the voltage dependence of steady-state fast inactivation, inconsistent temperature induced modulations were described in literature. Zimmermann et al. (2007), Ruff (1999), Ye et al. (2018), and Abdelsayed et al. (2013) reported significant hyperpolarizing shifts of fast inactivation *V*_*1/2*_ for Na_v_1.2, Na_v_1.4, Na_v_1.6, Na_v_1.7, and Na_v_1.8 with increasing temperature, while Egri et al. (2012) and Xiao et al. (2019) found no or only insignificant shifts for Na_v_1.2 and Na_v_1.8.

### Temperature intensifies the effects of pain-linked mutations

Hyperexcitability induced by increased temperature is also reflected in the phenotype of patients suffering from IEM, who experience pain attacks that can be triggered by mild warmth (van Genderen et al., 1993; Albuquerque et al., 2011). Except for two, all known IEM mutations so far go along with hyperpolarizing shifts in the *V*_*1/2*_ of activation (Baker and Nassar, 2020; Choi et al., 2010; Eberhardt et al., 2014). On comparing the activation *V*_*1/2*_ of Na_v_1.7/L823R with Na_v_1.7/WT, we observed a hyperpolarizing shift of ∼10 mV at all temperatures tested (Fig. 6, B and F). The additional shift of *V*_*1/2*_ to more negative potentials, especially at 35°C, may cause neuronal hyperexcitability leading to pain sensation. Moreover, cooling leads to a depolarizing shift, bringing the midpoint of activation from L823R closer to the one of WT. Similar temperature-induced effects were observed for the IEM mutation Na_v_1.7/L858F, which exhibited a depolarizing shift of activation *V*_*1/2*_ upon cooling to 16°C (Han et al., 2007). This effect is in line with reports that pain can only be alleviated by cooling for most IEM patients (van Genderen et al., 1993; Albuquerque et al., 2011; Tang et al., 2015). While the body core temperature is stably held at ∼37°C, the physiological temperature of the skin is ∼33°C–34°C (Miland and Mercer, 2006). Already a 1-min immersion into 15°C cold water can reduce it to 17°C (Dupuis, 1987) and longer immersion into ice-cooled water can drop the temperature of the fingers below 10°C (Isii et al., 2007). Thus, it is realistic that Na_v_1.7 channels that accumulate distally in nerve terminals of the skin are exposed to a large temperature range, and the skin can easily reach 15°C or even cooler temperatures.

The steady-state fast inactivation *V*_*1/2*_ of the IEM-linked Na_v_1.7/L823R was not shifted compared with WT at 25°C and 35°C, while at 15°C, a significant shift to more hyperpolarized potentials occurred (Fig. 6, C and G). This decreases the window current and thereby decreases the channel’s excitability at colder temperatures, which is in line with the clinical picture of IEM patients and the fact that cooling brings relief from pain. Ramp currents, on the other hand, were significantly enhanced compared with WT at every temperature tested for the IEM mutation, with the largest current occurring at 15°C. This suggests that ramp currents do not only depend on the window current but are probably strongly modulated by closed-state inactivation (Cummins et al., 1998; Estacion and Waxman, 2013). Like open-state inactivation, this seems to be slowed with decreasing temperature, leading to an increase in ramp current. The voltage at which peak ramp currents occur was, similar to activation *V*_*1/2*_, shifted to more depolarized potentials with decreasing temperature.

In patch-clamp experiments at room temperature, Lampert et al. (2009) also observed a shift of inactivation *V*_*1/2*_ of the mutant compared with WT. There, the search for a suitable explanation remained challenging because seen alone this would render the channel less excitable. This example points out that it is essential to perform electrophysiological investigations at different temperatures to understand the channel’s function in context and not to miss important effects.

In PEPD, patients also suffer from pain attacks, but in their case, cold wind is reported as a possible trigger factor (Fertleman et al., 2007). In contrast to the IEM mutation, a shift of activation of ∼9 mV to more depolarized potentials compared with WT was observed for the PEPD mutation Na_v_1.7/I1461T (Fig. 6, D and F). In literature, no significant shift in activation *V*_*1/2*_ compared with WT was reported before for Na_v_1.7/I1461T, but PEPD causing mutations in the segment 4 (S4)–S5 linker of DIII, Na_v_1.7/V1298F, and V1299F, as well as the mutation Na_v_1.7/T1464I showed shifts of 6.3, 4.5, and 6.8 mV, respectively, to more depolarized potentials (Fertleman et al., 2006; Jarecki et al., 2008). This is counterintuitive because the shift would render cells expressing the channels less excitable when focusing on this gating mode only. We assume that the impaired inactivation that was observed for I1461T is sufficient to overcome the predicted reduction in excitability induced by the shift of activation and is also the crucial factor for the special sensitivity at colder temperature.

10 of the 13 mutations causing PEPD that have been described so far are located in DIII and IV (Baker and Nassar, 2020). DIV plays an essential role in channel’s fast inactivation (McPhee et al., 1998; Capes et al., 2013; Goldschen-Ohm et al., 2013). Recently, Pan et al. (2018) proposed that rather than via direct occlusion, like is described with the “hinged lid” mechanism, fast inactivation may occur because of an allosteric mechanism. The IFM motif binds to a hydrophobic pocket, thereby causing a movement of DIV S6 toward the ion permeation pathway leading to occlusion. The hydrophobic cavity, to which the IFM motif binds, is formed by the S5 and S6 of DIII and IV and the DIII S4–5 linker (Pan et al, 2018; Shen et al, 2019). For the PEPD mutation Na_v_1.7/A1632E, located in DIV S5, the all-atom molecular dynamics simulations revealed that the glutamate side chain protrudes into the binding pocket and causes steric repulsion of the IFM motif. Thereby, it leads to impaired binding and thus impaired inactivation (Rühlmann et al., 2020). It is likely that changing the unpolar isoleucine of the IFM-motif in the Na_v_1.7/I1461T with a polar threonine results in a similar hydrophobic mismatch with impaired binding of the IFM into the hydrophobic cavity and a dysfunctional inactivation process. This is supported by the increase in *E*_*a*_ (Fig. 8 F) as well as the increase in persistent current (Fig. 6 A) of Na_v_1.7/I1461T compared with WT.

We observed that an ∼10 mV depolarizing shift in steady-state fast inactivation of Na_v_1.7/I1461T at 15°C and 25°C compared with WT was even larger at 35°C. This increases the window current, rendering neurons carrying the mutation more excitable (Fig. 6, E and G). Our results confirm the depolarizing shift of fast inactivation that has previously been described for Na_v_1.7/I1461T (Fertleman et al., 2006; Jarecki et al., 2008, 2009, 2010) and for several other PEPD-causing mutations at room temperature (Fertleman et al., 2006; Jarecki et al., 2008; Baker and Nassar, 2020; Dib-Hajj et al., 2008).

Temperature variations are also supposed to influence the Na_v_ gating kinetics. Here, we found an accelerated opening velocity, reflected in faster time-to-peak values observed with increasing temperature, similar to previous results (Touska et al., 2018; Almog et al., 2022). A faster opening could also promote increased excitability at elevated temperatures. Even though the L823R mutation induces an additional positive charge in the voltage sensor of DII, the activation kinetics were slightly slower than for WT. This effect was observed before and it was stated that it might be induced by an unusual rearrangement during the opening process caused by the extra arginine, with DII moving prior to DIII and thus increasing the time needed for opening (Lampert et al., 2009).

Temperature-sensitive epilepsy mutations in Na_v_1.1, causing febrile seizure, have shown different mechanisms causing hyperexcitability. While a lot of the responsible mutations in Na_v_1.1 are loss of function mutations, there are also some gain of function mutations described. Different from what was observed here for the IEM mutation, these mutations did not show hyperpolarizing shifts in activation explaining the hyperexcitability, but enlarged persistent currents when increasing the temperature to 37°C or more (Gorman et al., 2021; Jones et al., 2021; Peters et al., 2016; Volkers et al., 2013). An in-depth evaluation and comparison of these mutations could help to understand the structure-function relation during changing temperature.

### Subtype-specific modulation of inactivation kinetics

Our study revealed that Na_v_ inactivation kinetics are slowed with decreasing temperature resulting in a larger value of the inactivation time constant, similar to what has been reported by several others before (Zimmermann et al., 2007; Thomas et al., 2009; Ke et al., 2017; Sittl et al., 2012; Egri et al., 2012; Touska et al., 2018). The comparison of different subtypes, which was possible under standardized conditions in this study, showed that even though cooling has the same overall effect on all investigated Na_v_s, the extent of the effects differs strongly among subtypes, especially when focusing on the persistent current (Fig. 4). Compared with Na_v_1.7/WT at 15°C, Na_v_1.3 had more than eight times larger persistent current and a four times slower inactivation, and the high persistent current nearly diminished at 35°C. Differences in inactivation kinetics among the other subtypes were considerably smaller. This suggests Na_v_1.3 might have an important role in mediating cold or cold-related sensations.

Interestingly, we observed enhanced persistent current at 15°C and 25°C for Na_v_1.7/L823R compared with WT (Fig. 7). Persistent current of L823R was mainly detected in the voltage range between −70 and −30 mV, where not all channels are activated yet. Persistent currents in this voltage range might be related to the impaired balance of activation and inactivation, induced by the shift of activation to more hyperpolarized potentials, while steady-state fast inactivation *V*_*1/2*_ is at the same time nearly unaffected, but strongly slowed down by lowered temperature. In contrast, the large persistent current of Na_v_1.7/I1461T at 15°C is probably caused by an impaired inactivation mechanism.

### Thermodynamic analysis

With the Arrhenius analysis of the inactivation time constant *τ*, an estimation of the activation energy *E*_*a*_ for the transition from the open to the inactivated state was possible. Averaged values of *E*_*a*_ at potentials more depolarized than −10 mV were between 50 and 55 kJ/mol for Na_v_1.5, Na_v_1.6, Na_v_1.7, and Na_v_1.7/L823R. Na_v_1.3 and Na_v_1.7/I1461T displayed a clearly higher *E*_*a*_ with ≈79 and ≈78 kJ/mol (Figs. 4 and 8). This may explain the drastically slowed inactivation and the large persistent current observed at 15°C for these two channels. More energy is needed to inactivate them properly, and it is lacking at colder temperatures. Interestingly, Na_v_1.6 as well as both mutations showed no voltage dependence of *E*_*a*_, while the other subtypes did so. This indicates that the forward rate constant α and the backward rate constant β have the same *E*_*a*_ over the whole voltage range and not only at depolarized potentials.

### Enhanced excitability at cold temperatures as a result of persistent current and a potential role of resurgent current

A slowed or destabilized inactivation and large persistent current may lead to an increased resurgent current (Hampl et al., 2016). Current clamp experiments as well as computer models have linked resurgent currents to neuronal hyperexcitability (Jarecki et al., 2010; Sittl et al., 2012; Xiao et al., 2019). An increase in resurgent current has been observed for Na_v_1.7/I1461T and other PEPD mutations as well as other Na_v_ mutations slowing fast inactivation kinetics, and a correlation between the current decay time constant and resurgent current has been revealed (Jarecki et al., 2010; Theile et al., 2011; Sittl et al., 2012; Xiao et al., 2019; Hampl et al., 2016; Eberhardt et al., 2014). According to this, we assume a possible increase in resurgent current not only for the I1461T mutation but also for Na_v_1.3 at 15°C. Experiments with Oxaliplatin-treated nerve fibers revealed Na_v_1.6 mediated enhanced persistent and resurgent currents that were linked to cold-aggravated neuropathy (Sittl et al., 2012).

Taken together, a strongly slowed inactivation and thus enhanced persistent current can be induced by either a mutation (like for Na_v_1.7/I1461T) or cooling temperature (like for Na_v_1.3) or both, making it a potentially general mechanistic basis for cold aggravated symptoms and a possible explanation for the cold-induced hyperexcitability as potentially observed in patients suffering from PEPD. Cold-sensitive mutations in Na_v_1.4 causing PMC also support this hypothesis. PMC induces muscle stiffness in response to lowered ambient temperature. Several PMC mutations were shown to shift *V*_*1/2*_ of fast inactivation to more depolarized potentials, slow the current decay time constant, and accelerate the recovery from fast inactivation, similar to PEPD mutations (Bouhours et al., 2004; Farinato et al., 2019; Palmio et al., 2017; Ke et al., 2017). Na_v_1.1 plays an important role in TRPM8-positive Vglut3^lineage^ DRG-neurons, where it is resistant to entry into slow inactivation and seems to drive action potential firing (Griffith et al., 2019). Thus, broadening the investigation to other subtypes, such as Na_v_1.1 at lowered temperatures (e.g., 15°C), is likely to add important information for understanding a potential role in cold thermosensation.

### Na_v_1.3 overexpression may be crucial for cold allodynia after nerve injury

Na_v_1.3 exhibited a special sensitivity toward cooling compared with the other investigated subtypes, with exclusively large persistent currents and slow inactivation kinetics (Fig. 3, D and E). It is primarily expressed during the development of the nervous system, but it is expressed in only small amounts in adult DRG neurons (Felts et al., 1997; Chang et al., 2018). It was shown that Na_v_1.3 overexpression occurs in rat DRG neurons after peripheral axotomy (Dib-Hajj et al., 1996) or spinal cord injury (SCI; Hains et al., 2003; Lampert et al., 2006), leading to hyperexcitability of DRG nociceptive neurons and pain-related behavior due to rapidly repriming TTX-sensitive currents. Dorsal horn neurons of rats with SCI displayed enhanced persistent and ramp currents, supporting hyperexcitability (Lampert et al., 2006). Lindia et al. (2005) showed a higher sensitivity toward cold stimuli in rats after SCI compared with unaffected controls. Our results indicate that cold sensitivity after SCI may be explained by the overexpression of Na_v_1.3, whose inactivation kinetics are slowed by cooling, thus potentially leading to enhanced resurgent currents and hyperexcitability at low temperatures. Even though we carried out the experiments in HEK expressing rNa_v_1.3—not neuronal cells—and differences in the inactivation kinetics have been described for rNa_v_1.3 compared with hNa_v_1.3 (Tan and Soderlund, 2009), our results suggest that Na_v_1.3 overexpression may be a determining factor in the development of cold allodynia. Further investigation on the role of Na_v_1.3 in neuropathic pain is needed, such as exploring its potential as a candidate as a therapeutic target in the therapy of cold allodynia.

### The recovery from fast and the onset of slow inactivation is accelerated at 35°C

For all of the investigated Na_v_-WT channels as well as the Na_v_1.7/L823R mutation, our investigations revealed an acceleration of the recovery from inactivation when increasing the temperature from 15°C to 25°C. The rate of fast inactivation recovery increases even more at 35°C, but only for recovery times of 3–12 ms (depending on the subtype), and the fast recovering proportion of channels (% τ_*fast*_) decreases (Figs. 5 and 9; and Tables 1 and 2). With longer recovery periods, the recovery rate was smaller at 35°C compared to 25°C, suggesting that more channels are recovering slowly from inactivation with increasing temperature. We assume that with the 500 ms prepulse, which we used in our experiments, a slow inactivation component was detected in the investigation of recovery from fast inactivation at 35°C, reflected in the unexpected curve shapes and the high percentage of channels slowly recovering. This is consistent with the observation of a faster onset of slow inactivation with elevated temperature reported in the literature (Ke et al., 2017; Egri et al., 2012).

Taken together, our results suggest that with increasing temperature, the recovery from fast inactivation is accelerated, but at the same time, the entry into slow inactivation seems to be enhanced. This may result in higher availability of channels in high-frequency firing neurons at 35°C compared to 25°C and 15°C, but it would also protect Na_v_s from excessive firing resulting in hyperexcitability.

At 15°C, Na_v_1.7/I1461T was recovering more quickly, and use-dependent current decay was reduced compared with WT (Fig. 9 A). Thus, neurons carrying the mutation are more likely to show high-frequency firing at colder temperatures than neurons expressing the WT channel, an effect that was described at room temperature before (Jarecki et al., 2008; Sheets et al., 2011). Compared with WT, the I1461T mutation started to show a significantly slower recovery from fast inactivation for recovery-pulse durations longer than 10 ms, resulting in a flattening of the curve already at 25°C, while for the WT this effect was only observed at 35°C (Fig. 9 B). At this temperature, the mutated Na_v_s recovered mainly slowly, with a % τ_*fast*_ of only 37%, indicating that the proportion of channels recovering slowly was even more enhanced at this temperature. Even though Jarecki et al. (2008) described a decrease in the voltage-dependent transition into slow inactivated states for Na_v_1.7/I1461T and Sheets et al. (2011) observed no differences in the development of slow inactivation for WT and mutation, our observations suggest that this might be changed with increasing the temperature to 25°C or more. Further studies on the onset as well as recovery from fast inactivation are for sure necessary to get deeper insights into the mechanisms and temperature-induced effects. However, in combination with the results of the inactivation time constant and the enhanced persistent currents at 15°C, these results reinforce the special sensitivity of the I1461T mutation toward temperature changes in both directions and give evidence that neuronal hyperexcitability causing pain in the context of PEPD may be inducible by cooling.

### Putative role of the β1-subunit

The impact of β-subunits on the gating of Na_v_s should not be neglected in the discussion of temperature-induced changes in excitability. Next to the effect of β-subunits on the voltage dependence of gating states, resurgent currents (Grieco et al., 2005), stabilization against mechanical stimuli (Körner et al., 2018), and a possible contribution to intercellular communication and recruitment of Na_v_s (Malhotra et al., 2000), Egri et al. (2012) revealed a thermoprotective role of the β1-subunit. Temperature-induced changes to excitability are modulated by the expression of the β1-subunit with the general trend that at elevated temperature channels without β1 spend less time in the inactivated state. Thus, mutations in β1 can also lead to hyperexcitability at increased temperature, like was shown for the mutation β1(C121W), causing epilepsy with febrile seizures plus, a pediatric febrile seizure syndrome.

### Future investigations and outlook

Automated patch-clamp systems can nowadays perform high-throughput electrophysiological experiments and can at the same time adjust the temperature in a range from 10°C to 43°C. Compared with manual patch-clamp, where measurements at lowered or especially elevated temperatures are often complicated by technical obstacles, performing experiments at different temperatures is simplified by an automated patch-clamp device as it cools down or heats up not only the solutions but also the surroundings of the patched cells and thus produces a higher success rate compared with manual patch clamp. Nevertheless, the success rate declines with higher temperatures in both manual and automated patch clamps, whereas in the latter, this is potentially compensated by a higher number of parallelly recorded cells. However, with the automated patch device used for the here presented experiments, it was not possible to measure slow inactivation in a similar manner as with manual patch clamp due to cache overflow during the very long prepulses of more than 30 s. Most likely, this limitation will soon be overcome with the next technical developments. Automated patch clamp allows to increase the amount of obtained data by a multiple, while their quality remains comparable with those of manual patch clamp, with seal resistances above 1 GΩ that were maintained over time periods up to 15 min during the experiments. A limitation of automated patch clamp is the somewhat higher series resistances, which leads to a higher number of insufficiently clamped cells, which again can be compensated by the higher number of recordings (compare methods). At 35°C, we sometimes observed higher leak currents that weren’t subtracted properly (Fig. 1) and large sodium currents of more than 10 nA led to some inaccuracies in series-resistance compensations. With stable cell lines, on the other hand, we were able to achieve success rates of more than 80%. We chose 35°C as the highest temperature for recordings (not 37°C, which may have been more physiological for centrally expressed sodium channels) to guarantee reliable and reproducible high-quality recordings with an acceptable success rate.

For drug testing and safety pharmacology, it is crucial to gain deeper insight into gating mechanisms at the physiological temperature of different ion channels. The state dependence of drug binding is likely to be coupled with the occupancy of different conformational states of the channel (Sheets et al., 2011), and this is in turn dependent on temperature. E-4031, an experimental class III antiarrhythmic drug, was for example fivefold more potent in blocking hERG channels at 35°C than at 22°C (Davie et al., 2004). Thus, to investigate accurate potencies of drugs that modulate ion channels, it would be preferable to perform these studies at 37°C.

Detailed electrophysiological investigation of Na_v_s at different temperatures is also essential to understand the properties and to gain a deeper insight into the complex gating mechanisms of Na_v_s at physiological temperature, otherwise important effects might be missed. Of course, in the physiological context, the excitability of a tissue does not only depend on one single channel but also the interplay between many different channels. Furthermore, passive membrane properties have been shown to be temperature sensitive and alter excitability (Touska et al., 2018; Volgushev et al., 2000). However, biologically detailed neuronal simulation models contain still crude assumptions about the kinetics of Na_v_s and many poorly constrained parameters, like Q10 values, that should only be used carefully because of their temperature and voltage dependence (Almog and Korngreen, 2016). In the future, the data collected in this work should be included in detailed neuronal computer models, based on Hodgkin-Huxley or Markov models. Because these models benefit from reliable data with a high *n*-number, our study provides a solid basis for these studies. With this, the predicted changes in neuronal excitability caused by the combination of mutation effects and temperature could be simulated and provide a deeper understanding. With the help of a computational model, the differences in excitability along sensory axons from the skin (32°C and lower) toward the spinal cord (∼37°C) could be addressed.

Thus, it is of enormous importance to collect experimental data of different ion channels also at physiological temperatures to improve the reliability of action potential simulations and neuronal models, draw (patho-)physiological relevant conclusions, and thus understand Na_v_s in their physiological context.

## Supplementary Material

Table S1 provides a ummary of cell culture media and supplements which were used for cultivation of HEK293 rNav1.3, hNav1.5, mNav1.6, hNav1.7/WT, hNav1.7/L823R, and hNav1.7/I1461T.

Table S2 shows voltage error and series resistance for all investigated Nav-subtypes.

## Data Availability

All data are available upon request.

## Data availability

All data are available upon request.
