# Supplementary material for: Cold and warmth intensify pain-linked sodium channel gating effects and persistent currents
Source: J Gen Physiol. 2023 Aug 2;155(9):e202213312. doi: 10.1085/jgp.202213312 (PMC10397059; doi:10.1085/jgp.202213312)
Supplement: Table S1 — provides a ummary of cell culture media and supplements which were used for cultivation of HEK293 rNav1.3, hNav1.5, mNav1.6, hNav1.7/WT, hNav1.7/L823R, and hNav1.7/I1461T. [file JGP_202213312_TableS1.docx]

**Table S1.** Summary of cell-culture media and supplements which were used for cultivation of HEK293 rNa_v_1.3, hNa_v_1.5, mNa_v_1.6, hNa_v_1.7/WT, hNa_v_1.7/L823R, and hNa_v_1.7/I1461T

| **Stable cell-line** | **Cell-culture media and supplements** |
| --- | --- |
| **HEK293 rNa_v_1.3**  (Cummins et al., 2001) | Dulbecco´s modified Eagle medium (DMEM) with 4,5 % Glucose and L-Glutamine (Thermo Fisher Scientific, Waltham, Massachusetts, USA)  10 % fetal bovine serum (FBS; Sigma-Aldrich, St. Louis, Missouri, USA)  0,5 mg/ml Genetecin (G418; Carl Roth GmbH+Co.KG, Karlsruhe, Germany) |
| **HEK293 hNa_v_1.5**  (Eberhardt et al., 2015) | Dulbecco´s modified Eagle medium F-12 (DMEM/F1-2; Thermo Fisher Scientific, Waltham, Massachusetts, USA), 10 % FBS, 100 µg/ml Zeocin (Invivogen, San Diego, California, USA) |
| **HEK293 mNa_v_1.6**  (Herzog et al., 2003, Laezza et al., 2009) | DMEM/F-12, 10 % FBS, 0,5 mg/ml G418 |
| **HEK293 hNa_v_1.7**  (From Anaxon AG, Berne, Switzerland) | Ham´s F-12 Nutrient Mix, GlutaMAX^TM^ Supplement (Thermo Fisher Scientific, Waltham, Massachusetts, USA), 9 % FBS, 1 mM Sodium Pyruvate (Thermo Fisher Scientific, Waltham, Massachusetts, USA), 150 µg/ml Hygromycin B (Carl Roth GmbH+Co.KG, Karlsruhe, Germany) |
| **HEK293 hNa_v_1.7/L823R**  (Merck KGaA, Darmstadt, Germany) | DMEM, 10 % FBS, 0,1 mM MEM Non-Essential Amino Acids Solution (NEAA; Thermo Fisher Scientific, Waltham, Massachusetts, USA), 25 mM HEPES (Carl Roth GmbH+Co.KG, Karlsruhe, Germany), 1 mg/ml G418, 5 µg/ml Blasticidin (Thermo Fisher Scientific, Waltham, Massachusetts, USA)  (1 µg/ml Doxycycline (Sigma-Aldrich, St. Louis, Missouri, USA) added 24 hours prior to the experiment) |
| **HEK293 hNa_v_1.7/I1461T**  (Merck KGaA, Darmstadt, Germany) | DMEM, 10% FBS, 0,1 mM NEAA, 25 mM HEPES, 10 µg/ml Blasticidin |

CUMMINS, T. R., AGLIECO, F., RENGANATHAN, M., HERZOG, R. I., DIB-HAJJ, S. D. & WAXMAN, S. G. 2001. Nav1.3 sodium channels: rapid repriming and slow closed-state inactivation display quantitative differences after expression in a mammalian cell line and in spinal sensory neurons. *J Neurosci,* 21**,** 5952-5961.

EBERHARDT, E., HAVLICEK, S., SCHMIDT, D., LINK, ANDREA S., NEACSU, C., KOHL, Z., HAMPL, M., KIST, ANDREAS M., KLINGER, A., NAU, C., SCHÜTTLER, J., ALZHEIMER, C., WINKLER, J., NAMER, B., WINNER, B. & LAMPERT, A. 2015. Pattern of Functional TTX-Resistant Sodium Channels Reveals a Developmental Stage of Human iPSC- and ESC-Derived Nociceptors. *Stem Cell Reports,* 5**,** 305-313.

HERZOG, R. I., CUMMINS, T. R., GHASSEMI, F., DIB-HAJJ, S. D. & WAXMAN, S. G. 2003. Distinct repriming and closed-state inactivation kinetics of Nav1.6 and Nav1.7 sodium channels in mouse spinal sensory neurons. *The Journal of physiology,* 551**,** 741-750.

LAEZZA, F., LAMPERT, A., KOZEL, M. A., GERBER, B. R., RUSH, A. M., NERBONNE, J. M., WAXMAN, S. G., DIB-HAJJ, S. D. & ORNITZ, D. M. 2009. FGF14 N-terminal splice variants differentially modulate Nav1.2 and Nav1.6-encoded sodium channels. *Molecular and Cellular Neuroscience,* 42**,** 90-101.
