# Supplementary material for: Cold and warmth intensify pain-linked sodium channel gating effects and persistent currents
Source: J Gen Physiol. 2023 Aug 2;155(9):e202213312. doi: 10.1085/jgp.202213312 (PMC10397059; doi:10.1085/jgp.202213312)
Supplement: Table S2 — shows voltage error and series resistance for all investigated Nav-subtypes. [file JGP_202213312_TableS2.docx]

|  | **Voltage error (mV)** | | | **Series resistance (MΩ)** | | |
| --- | --- | --- | --- | --- | --- | --- |
|  | ***15 °C*** | ***25 °C*** | ***35 °C*** | ***15 °C*** | ***25 °C*** | ***35 °C*** |
| Na_v_1.3 | -11.4 ± 1.2 | 0.5 ± 0.7 | -4.4 ± 1.2 | 7.0 ± 0.4 | 5.1 ± 0.4 | 6.0 ± 0.6 |
| Na_v_1.5 | 0.95 ± 0.5 | 6.1 ± 0.9 | -0.01 ± 1.5 | 6.5 ± 0.7 | 4.2 ± 0.3 | 5.5 ± 0.9 |
| Na_v_1.6 | 7.5 ± 0.8 | 10.8 ± 1.2 | -2.7 ± 4.1 | 7.2 ± 0.7 | 5.6 ± 0.9 | 6.6 ± 0.7 |
| Na_v_1.7 | -0.7 ± 0.6 | 0.3 ± 0.8 | -10.0 ± 1.3 | 6.3 ± 0.5 | 5.9 ± 0.4 | 5.5 ± 0.5 |
| Na_v_1.7/L823R | -3.4 ± 1.2 | 2.2 ± 0.7 | -8.4 ± 2.6 | 4.9 ± 0.3 | 4.2 ± 0.2 | 5.0 ± 0.5 |
| Na_v_1./I1461T | -13.4 ± 2.6 | -8.3 ± 1.7 | -26.2 ± 3.3 | 6.0 ± 0.5 | 4.2 ± 0.4 | 3.8 ± 0.3 |

**Table S2.** Voltage error and series resistance for all investigated Na_v_-subtypes. Data presented as mean ± SEM. n values are listed in table 1 (activation V_1/2_).
